# Flexible Players within the Sheaths: The Intrinsically Disordered Proteins of Myelin in Health and Disease

**DOI:** 10.3390/cells9020470

**Published:** 2020-02-18

**Authors:** Arne Raasakka, Petri Kursula

**Affiliations:** 1 Department of Biomedicine, University of Bergen, Jonas Lies vei 91, NO-5009 Bergen, Norway; arne.raasakka@uib.no; 2 Faculty of Biochemistry and Molecular Medicine & Biocenter Oulu, University of Oulu, Aapistie 7A, FI-90220 Oulu, Finland

**Keywords:** myelin, intrinsically disordered protein, multiple sclerosis, peripheral neuropathies, myelination, protein folding, protein–membrane interaction, protein–protein interaction

## Abstract

Myelin ensheathes selected axonal segments within the nervous system, resulting primarily in nerve impulse acceleration, as well as mechanical and trophic support for neurons. In the central and peripheral nervous systems, various proteins that contribute to the formation and stability of myelin are present, which also harbor pathophysiological roles in myelin disease. Many myelin proteins have common attributes, including small size, hydrophobic segments, multifunctionality, longevity, and regions of intrinsic disorder. With recent advances in protein biophysical characterization and bioinformatics, it has become evident that intrinsically disordered proteins (IDPs) are abundant in myelin, and their flexible nature enables multifunctionality. Here, we review known myelin IDPs, their conservation, molecular characteristics and functions, and their disease relevance, along with open questions and speculations. We place emphasis on classifying the molecular details of IDPs in myelin, and we correlate these with their various functions, including susceptibility to post-translational modifications, function in protein–protein and protein–membrane interactions, as well as their role as extended entropic chains. We discuss how myelin pathology can relate to IDPs and which molecular factors are potentially involved.

## 1. Introduction

The vertebrate nervous system has evolved to serve a vast diversity of animals, including humans. The brain and the spinal cord form the central nervous system (CNS), which orchestrates information storage and processing, as well as reads sensory output. On the other hand, the peripheral nervous system (PNS) acts as a vital link between the CNS and peripheral organs. While the nervous system is well-organized in terms of nerve tracts, neurons lack sufficient action potential propagation efficiency due to their relatively small diameter (usually 0.1–20 µm) compared to their length (up to ~1 m) [1,2,3]. Another limitation is the availability of metabolic energy, as sustained nerve impulse firing is an active process that requires ATP—much of which is obtained through mitochondrial activity [4]. The giant squid has solved the efficiency problem through the evolution of giant axons—some up to 1 mm in diameter [5]—but in vertebrates, another solution allows the acceleration of nerve impulses up to 100-fold: axon insulation by myelin.

**Figure 1:**
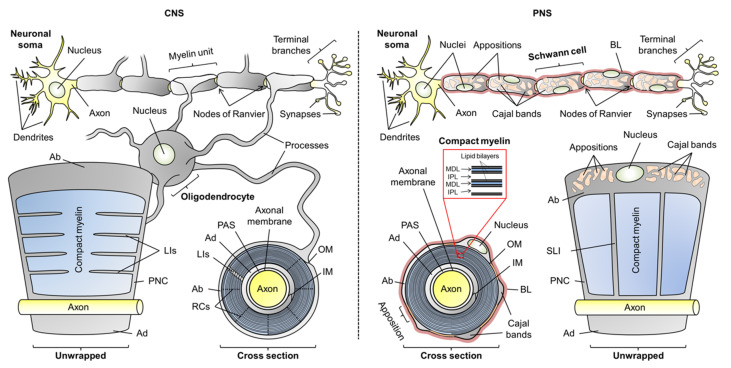
The anatomy of oligodendrocytic and Schwann cell myelin sheaths in the central nervous system (CNS) and peripheral nervous system (PNS), respectively. The arrangement of multiple myelin units along an axon is illustrated, as well as unwrapped myelin units and cross sections. Compact myelin and non-compact myelin are colored blue and gray, respectively. Abbreviations: Ab, abaxonal layer; Ad, adaxonal layer; BL, basal lamina; IM, inner mesaxon; IPL, intraperiod line; LIs, longitudinal incisures; MDL, major dense line; OM, outer mesaxon; PAS, periaxonal space; PNC, paranodal collar; RCs, radial components; SLI, Schmidt–Lanterman incisures.

Myelin is a specialized plasma membrane produced by myelinating glia: oligodendrocytes in the CNS and Schwann cells in the PNS. The myelin membrane is wrapped around axons tens of times and compacted in a process driven by actin disassembly and membrane stacking via abundant adhesion proteins [6,7]. The outcome is a lipid-rich sheath with low water content, separating the axonal surface from the extracellular milieu. In the CNS, oligodendrocytes grow long processes that form single myelin units; however, a single oligodendrocyte can myelinate several axons. In the PNS, each Schwann cell wraps around an axon and forms one myelin unit (Figure 1). Individual myelin units along the axon are separated by nodes of Ranvier, where the axonal membrane is rich in voltage-gated ion channels. Electrical insulation and the arrangement of ion channel clusters form the basis of saltatory conduction [8].

The overall morphology of myelin is similar in the CNS and PNS, but there are notable ultrastructural differences. Most of the myelin sheath is compact myelin—tightly stacked proteolipid membrane multilayers with low water content. This promotes the insulative character of myelin. Non-compact myelin lines the outer- and innermost layers of myelin, known as the abaxonal and adaxonal layers, respectively. Additionally, it forms paranodal loops—structures at the ends of the myelin unit that anchor it to the axon. Cytoplasmic channels that traverse through compact myelin in the PNS and CNS are known as Schmidt–Lanterman incisures and longitudinal incisures, respectively. Water is abundant in non-compact myelin, which contains cytoskeletal elements and serves as a maintenance compartment in the myelin sheath (Figure 1) [4,9].

In the PNS, the myelin sheath is surrounded by a carbohydrate-rich basal lamina; such a structure is not present in CNS myelin [10]. Additionally, the abaxonal space of Schwann cells is partially compacted to so-called membrane appositions, which line veins of cytoplasm known as Cajal bands. Membrane appositions and Cajal bands are required for the correct function of PNS myelin, but the role of membrane appositions is poorly understood [11,12,13]. The space that separates the adaxonal membrane from the axonal membrane is the periaxonal space, in which axoglial signaling and adhesion take place [14].

The narrow extracellular space between the periodic compact myelin membranes is called the intramyelinic compartment. The lipid-rich myelin membrane carries a high content of cholesterol, which is essential for myelination [15,16]. The myelin membrane is asymmetric: the extracellular/intramyelinic monolayer is rich in glycolipids, whereas the cytoplasmic leaflet is predominantly formed of phospholipids and harbors a net negative charge [17]. This charge is one of the main driving factors of protein–lipid interactions in myelin [18,19,20,21,22,23,24]. In compact myelin, several proteins contribute to the stacking of lipid bilayers, which form a highly periodic arrangement that can be characterized using X-ray and neutron scattering [25,26]. The nearly fused cytoplasmic leaflets of compact myelin were visible already in early electron micrographs as repetitive electron-dense features, and they were thus named the major dense lines (MDL). The alternating intramyelinic compartment was named the intraperiod line (IPL) [27,28,29].

The packing of compact myelin is so tight that it excludes the presence of most proteins [30]. The proteins of myelin are often specific to the myelin sheath and multifunctional. They are related to the development of myelin diseases, such as multiple sclerosis (MS) and peripheral neuropathies. The lipid-rich nature of myelin and its narrow compartments make it a particularly challenging system to study, which is the main reason why myelin proteins and their role in disease are undercharacterized. However, giant leaps in myelin protein research have been taken in recent years, especially in the study of myelin intrinsically disordered proteins (IDPs), which are the main focus of this review.

## 2. Intrinsically Disordered Proteins of Myelin

### 2.1. General Attributes of Myelin-Specific Proteins

Proteins in myelin come in many shapes and forms, but not sizes. While non-compact myelin contains both “typical” soluble and membrane proteins, compact myelin contains a mere handful of proteins, some specific to CNS or PNS, which are capped by one attribute over others: molecular weight. Most compact myelin proteins are smaller than 30 kDa in size [31,32]. Given the very narrow cytoplasmic and intramyelinic spacing in compact myelin [33,34], it comes as no surprise that only small proteins are present. A physical size barrier that limits the diffusion of large soluble domains has been proven to exist [30]. However, the small but abundant myelin proteins, such as protein zero (P0), have been described to undergo multimerization, although the details of these structural arrangements are currently unknown [35,36,37,38,39]. The myelin protein multimers are likely to involve interactions in the membrane plane, as well as between proteins present on apposing membranes, resulting in ordered 3D organization of myelin proteins and lipid bilayers.

**Figure 2:**
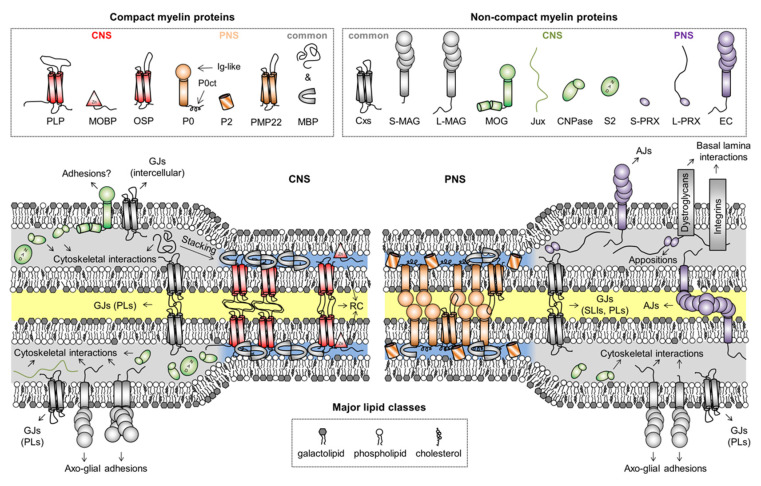
Myelin proteins and their compartmentalization. Some proteins are shared between CNS and PNS myelin, but their expression levels can vary drastically between the two, like in the case of CNPase, which is predominantly a CNS enzyme. The intracellular compartment is colored blue and gray for compact and non-compact myelin, respectively. The extracellular and intramyelinic compartments are colored white and yellow, respectively. Note that cytoskeletal elements, other common proteins, and cell organelles are not included for clarity. Abbreviations: AJs, adherens junctions; CNPase, 2′,3′-cyclic nucleotide 3′-phosphodiesterase; Cxs, connexins; EC, epithelial cadherin; GJs, gap junctions; Ig-like, immunoglobulin-like; Jux, juxtanodin; LIs, longitudal incisures; MAG, myelin-associated glycoprotein; MBP, myelin basic protein; MOBP, myelin-associated oligodendrocytic basic protein; MOG, myelin/oligodendrocyte glycoprotein; OSP, oligodendrocyte-specific protein/claudin 11; P0, myelin protein zero; P0ct, the cytoplasmic domain of P0; P2, peripheral myelin protein 2; PLP, proteolipid protein; PLs, paranodal loops; PMP22, peripheral myelin protein 22; PRX, periaxin; RC, radial component; S2, sirtuin 2; SLIs, Schmidt–Lanterman incisures. In MAG and PRX, the L- and S- prefixes indicate long and short isoforms, respectively.

The myelin sheath contains relatively few different proteins (Figure 2). The major myelin proteins present certain characteristics, such as extreme longevity [40], multifunctionality [41,42,43,44], full, partial, or transient binding to membranes, as well as strict localization to either compact or non-compact myelin, and further sub-localization to different ultrastructural compartments [45]. Despite decades of research, the functions and structure-function relationships of several myelin proteins have emerged only in recent years, and many open questions remain.

Illustrated in Figure 2 are proteins considered myelin-specific, categorized by their localization to the CNS or PNS, as well as to compact or non-compact myelin. The functions and structures of several myelin proteins have been discussed earlier [45,46,47,48,49,50], and here, we will focus on the IDPs of myelin. Some borderline cases of disorder do exist in the myelin proteome that will not be covered. These include the intra- and extracellular loops of various tetraspan membrane proteins, which are often predicted to be disordered [23]. However, these structures are very close to the myelin membrane and therefore likely to be folded, as experimentally shown for the loops of proteolipid protein [23]. Another example is the C-terminal extension of P0 (P0ct), which is disordered in the absence of lipids, but not under membrane-like conditions [19,51,52,53,54]. In vivo, the lipidated P0ct directly follows the transmembrane helix of P0 in the cytoplasmic compartment of PNS compact myelin, permanently anchoring it to the phospholipid membrane, whereby it folds and is thus unlikely to function as a canonical IDP [55].

General IDP categorization guidelines have been exhaustively discussed [56], which will serve as a fundamental basis in this review. When applicable, IDPs and disordered regions will be classified based on functional features into short linear motifs, molecular recognition features (MoRFs), and disordered domains. Sequence features and overall sequence composition will be used to understand and predict the functions of IDPs in myelin.

### 2.2. Myelin Basic Protein

**Figure 3:**
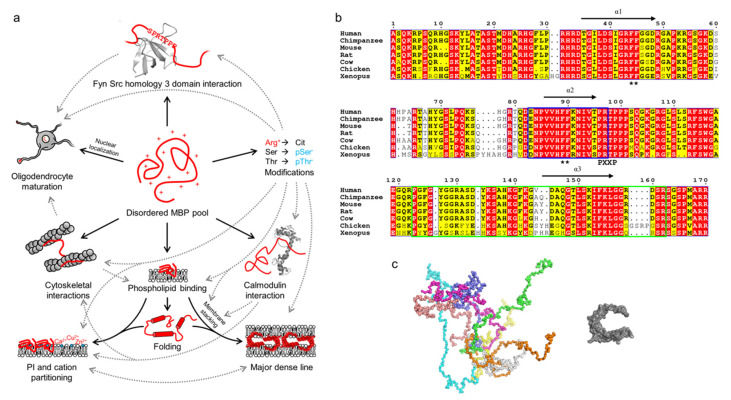
The multifunctionality, conformations, and conservation of MBP. (**a**) Schematic of the multifunctionality (solid arrows) of MBP, which arises through its disordered nature. Various PTMs, especially citrullination and phosphorylation, regulate the known functions of MBP (dashed arrows). The panel was inspired by Vassall et al. [65]. (**b**) Sequence alignment of 18.5-kDa MBP from vertebrates generated using ESPript [66]. MBP is highly conserved, especially all helically folding, lipid-interacting segments (black arrows; α1-α3), one of which overlaps with the immunogenic region (blue outlines). A noteworthy feature is the conservation of Arg residues, most of which are targets for citrullination. Black asterisks denote double-Phe motifs that are required for the phase transition of MBP upon lipid binding. Residue numbering corresponds to human MBP. (**c**) Conformational ensemble of 18.5-kDa MBP as determined using SAXS [24] (left) in comparison to a model of lipid-bound MBP [67] (right). Each colored chain in the ensemble represents a single conformational subpopulation in the pool of disordered MBP.

Myelin basic protein (MBP) is an archetypal IDP and one of the best-characterized proteins of the myelin sheath. For decades, the functional and structural aspects of MBP have been unraveled together with its possible involvement in MS, and an impressive amount of literature exists (see [41,45,57,58,59,60,61,62,63,64,65], for example). MBP is a multifunctional protein involved in a plethora of processes, from cytoskeletal interactions to the stacking of membrane multilayers in compact myelin (Figure 3a) [65].

MBP manifests itself as several isoforms that arise through alternative splicing [68,69]. These are divided into classical isoforms that are mostly present in the cytosol [70], and Golli isoforms that undergo nuclear localization and influence intracellular Ca^2+^ levels [71,72]. Especially the classical isoforms exist as a heterogeneous mixture in myelin and myelinating cells, although the 18.5-kDa isoform is predominant [31,32,59]. All MBPs are basic due to a high number of positively charged residues (Figure 3b), which translates to a high isoelectric point (pI) and a high positive net charge under physiological pH.

MBP is translated in the cytoplasm, especially during myelin compaction, where its translation occurs locally, when it is needed [64,73,74]. Some classical MBP isoforms, as well as non-classical Golli isoforms, localize to the nucleus, potentially harboring a role in oligodendrocytic differentiation. Interaction partners that would bind to nuclear MBPs are yet to be described [75,76,77].

MBP takes part in several protein–protein interactions, and thus acts as an effector. MBP interacts with Fyn kinase [78,79,80], cytoskeletal elements [78,81], and calmodulin, the latter interaction being dependent on Ca^2+^ [21,82,83,84]. The interaction with Fyn kinase is mediated via the SH3 domain, which MBP binds through a conserved PXXP motif. The interaction has a potential impact on oligodendrocytic differentiation, as Fyn signaling is important during myelin development [79,80]. Oligodendrocyte process growth is thought to be modulated by the interaction of MBP with the cytoskeleton [78,81], which in turn is affected by the interaction between MBP and calmodulin [21,85]. These interactions suggest, combined with potential effects of nuclear MBP, that MBP harbors a role in oligodendrocyte differentiation through several mechanisms. In addition to protein–protein interactions, MBP binds nucleotides and divalent cations [21,86,87,88]. The divalent cations Ca^2+^ and Zn^2+^ contribute to the membrane stacking of myelin [53,86,87,89,90] (see below).

MBP is an excellent example of an IDP that displays several sites for post-translational modifications (PTMs; Figure 3a) [91]. Ser/Thr phosphorylation and Arg deimination (citrullination) are the most abundant charge-modifying PTMs [60,92,93,94,95,96,97,98,99,100], the latter being irreversible. MBP citrullination is carried out in the cytoplasm by peptidyl–arginine deiminases [101]. This results in the eight charge isomers of MBP, C1–C8, that display an increasing degree of citrullination. C1 is uncitrullinated and the most basic isomer (+19 charge in physiological conditions), while C8 is the least basic isomer [59]. The significance of the several charge isomers is not entirely understood, although MBP deimination levels appear to follow myelin developmental stages [62]. The least basic C8 isomer has been shown to be unable to maintain the integrity of compact myelin [102], and to localize to the IPL in the CNS, whereas less modified MBP is predominantly present in the MDL [103]. Additionally, the actin interactions of C8 are subtly reduced, with its ability to connect actin with the myelin membrane being most affected [104]. In contrast to the C1 isomer, C8 lacks the ability to induce phospholipase C activity [105]. The functions and localization of different MBP charge isoforms are subject to future studies, especially in Schwann cells.

Phosphorylation regulates some functions of MBP, such as binding to Fyn tyrosine kinase [79,97]. MBP is highly conserved in vertebrates (Figure 3b). Notable conserved sites include the independently folding helical segments (see below), one of which contains an autoantigenic epitope [106,107]. MBP is rich in Arg residues, most of which are conserved in vertebrates, especially in mammals. Most Arg residues in the 18.5-kDa MBP are citrullination targets [60,92,93].

The best-characterized function of MBP is its ability to produce stable membrane stacks upon the formation of compact myelin (Figure 3a) [20,30,108]. The stacking is dependent on negatively charged lipids, especially phosphatidylinositol phosphates [90,109], other lipids (cholesterol, sphingomyelin, and phosphatidylethanolamines) [110,111,112], as well as ionic strength [18,53], divalent cations [53,86,87,89,90], the PTM state of MBP [21,113], and its interactions [21,104]. The negative net charge of the phospholipid membrane attracts MBP [24], which binds and partially folds in the process. A pre-stack intermediate state is formed, which displays elongated MBP as a surface that can adhere to an apposing membrane [24]. The final membrane stack forms through MBP undergoing a phase transition into a molecular glue [114], which has an amorphous structure in electron microscopy (EM) [24]. The MBP phase transition depends on two conserved double-Phe motifs (Figure 3b) [114]. Membrane-bound MBP has been found to segregate phosphatidylinositol phosphates and divalent cations [90,109]. Based on circular dichroism (CD) spectroscopy and small-angle X-ray scattering (SAXS) experiments, MBP in solution behaves like a random coil with distinct conformational subpopulations [24]. Upon lipid binding, MBP has been shown to fold into a C-shaped molecule based on EM, SAXS, and molecular modeling (Figure 3c) [67,115,116]. While the atomic-resolution details of MBP folding are not known, nuclear magnetic resonance (NMR) spectroscopy has revealed three segments in MBP that can fold into amphipathic α-helices under membrane-mimicking conditions (Figure 3a) [107,117,118]. In the formation of cytoplasmic channels in myelin, MBP-mediated membrane stacking works antagonistically against the binding of actin to membrane-associated 2′,3′-cyclic nucleotide 3′-phosphodiesterase (CNPase) [119,120].

### 2.3. Myelin-Associated Oligodendrocytic Basic Protein

Myelin-associated oligodendrocytic basic protein (MOBP) is a poorly characterized but notably abundant protein in CNS myelin [121]. Like MBP, MOBP is rich in cationic amino acids and exists as an array of splice isoforms [121,122]. Its function, however, is elusive [123]. MOBP was initially suggested to stack membranes like MBP, but it has since been shown to be involved in the formation of the radial component, a series of tight membrane junctions in CNS compact myelin [124,125,126].

Human MOBP has some interesting features that distinguish it from being an MBP-like basic protein. Firstly, it contains an N-terminal Cys-rich region predicted to be a FYVE-like zinc-finger domain, which most likely penetrates into a membrane after folding and Zn^2+^ binding [48]. Myelin has a rather high abundance of Zn^2+^, as well as other divalent cations [127,128], and Zn^2+^ has been linked to myelin pathophysiology (see below) [129,130]. Zn^2+^ might not be crucial for MOBP membrane binding, as peptides from the N-terminal domain fold in the presence of phospholipids [23]. Secondly, MOBP contains a C-terminal Pro-rich region that spans half of the 183-residue major isoform. This region partially consists of 10-residue tandem repeats with the sequence PRSPPRSERQ.

**Figure 4:**
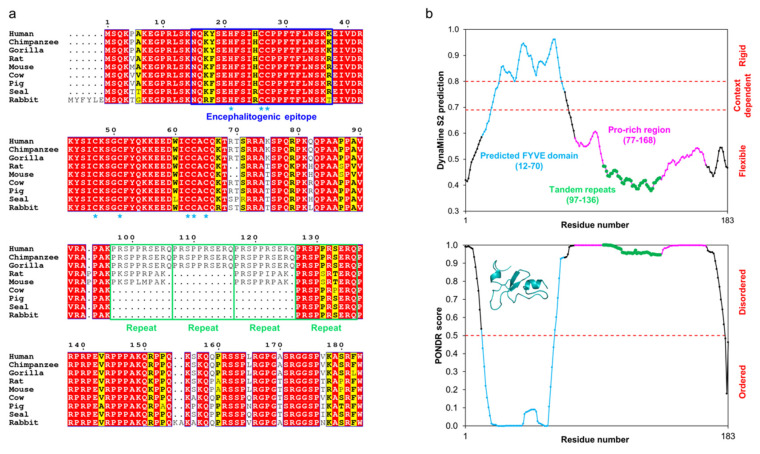
The conservation and predicted flexibility of MOBP isoform 1. (**a**) Sequence alignment of mammalian MOBPs generated using ESPript [66]. MOBP is highly conserved throughout mammals and especially within primates. Residue numbering corresponds to human MOBP. The residues predicted [48] to interact with Zn^2+^ in the putative FYVE domain have been indicated with blue asterisks. The tandem repeats within the Pro-rich region are indicated with green outlines. (**b**) DynaMine [132] (top) and PONDR [133] (bottom) predict human MOBP to be mostly disordered, with a folded N-terminal FYVE domain. The various compositional regions have been indicated. The structure in the PONDR inset represents the Phyre^2^ [134] prediction of the FYVE domain.

MOBP is highly conserved in mammals. However, the tandem repeat region differs in the amount of repeats between species (Figure 4a), is predicted to be very flexible (Figure 4b), does not fold in the presence of detergents or 30% TFE [55], and is unlikely to be folded under physiological conditions (Pro spacing suggests an entropic chain). With an abundance of Arg and Lys over Asp and Glu, MOBP is classified as a basic polyelectrolyte similarly to MBP. Therefore, it might interact with phospholipids in the narrow cytoplasmic compartment of the MDL, despite remaining unfolded in membrane-mimicking conditions [55]. Interestingly, the PTMs described for rat MOBP only include the phosphorylation of Ser85, Ser98, and Ser107 [131]. This apparently low number of PTMs might be explained by rapid confinement of MOBP into the MDL of mature myelin, whereas MBP can display its PTM sites as an unfolded chain, before it associates with membranes or carries out its other functions. More experimental evidence is needed to confirm MOBP-membrane interactions in myelin, as well as to map the full spectrum of PTMs in MOBP.

The tandem repeats are highly conserved, although the number of repeat units may vary between species (Figure 4a). All mammalian MOBPs accessible through BLAST that correspond to the canonical 183-residue human MOBP splice isoform have at least one of these regions, implying importance of this sequence. To date, no published structural data on full-length MOBP exist, let alone constructs that lack any or all tandem repeats in vitro. A recent study on the Fyn kinase-regulated translation of MOBP concluded that the N-terminal region of MOBP is involved in oligodendrocyte differentiation, while removal of the Pro-rich region has minimal impact on this [135]. The 81-residue splicing isoform of MOBP, which lacks the entire Pro-rich region and only has the FYVE domain, displays a prominent expression pattern in the early stages of myelin development [122]. Thus, the N- and C-terminal halves of MOBP may have different roles in myelin formation.

### 2.4. 2′,3′-Cyclic Nucleotide 3′-Phosphodiesterase

CNPase is a well-characterized enzyme of non-compact CNS myelin, where it makes up 4% of total myelin protein [32,44,136]. It is also present in minor amounts in the PNS [137]. The majority of CNPase localizes to the cytosol (isoform 1), but a small fraction is transported to mitochondria through an N-terminal targeting sequence (isoform 2) [138,139,140].

**Figure 5:**
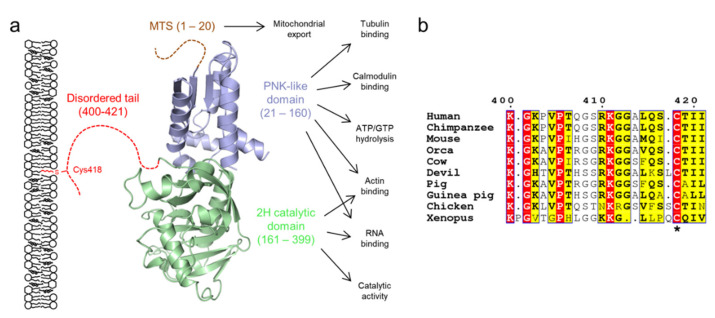
The domain structure of CNPase. (**a**) CNPase consists of two folded domains and a C-terminal 20-residue disordered tail, which mediates membrane interactions via the lipidated residue Cys418. Isoform 2 contains an additional N-terminal mitochondrial targeting sequence (MTS). The C-terminal tail tethers CNPase to the membrane, while it carries out its various functions [44,141]. (**b**) The C-terminal tail is conserved within several vertebrates, but is lost e.g. in fishes [141]. The lipidated Cys residue is indicated with an asterisk. Residue numbering corresponds to human CNPase. Orca, killer whale; Devil, Tasmanian devil; Xenopus, African clawed frog.

Mammalian CNPase has two structured domains followed by a disordered 20-residue C-terminal tail (Figure 5) [141]. The N-terminal polynucleotide kinase (PNK)-like domain is folded, and it possibly mediates CNPase homodimerization [142], interacts with calmodulin [143], and is able to bind and hydrolyze nucleoside triphosphates [144]. The 2*H* domain is the best-characterized region of CNPase, being responsible for its phosphodiesterase activity [145]. Its structure has been resolved at atomic resolution [146,147,148,149,150]. The enzymatic activity has been extensively characterized [142,145,146,148,149,150,151], and a potential pathway that gives CNPase a physiological role has been proposed [152,153,154]. In addition to catalytic activity, the folded domains of CNPase have other functions, including RNA [155], microtubule [156,157], and actin binding [120,158]. The latter enables CNPase to regulate the formation of cytoplasmic cavities in compact myelin together with MBP [120]. In mitochondria, CNPase has been linked to the opening of the transition pore complex, a proapoptotic mechanism resulting in Ca^2+^ release to the cytoplasm [159,160,161].

The C-terminal tail of CNPase has been experimentally characterized to be disordered using CD and SAXS [55,142,148,149,162], and it mediates membrane binding through a lipidated Cys residue [162]. When anchored to the oligodendrocyte plasma membrane, CNPase can bridge cytoskeletal elements to the membrane (Figure 5a) [156]. CD experiments demonstrate that the tail is mostly unfolded under membrane-mimicking conditions [55], which implies that the tail is likely to remain disordered when membrane-bound. On the other hand, the tail may turn towards the folded domains of CNPase [149], and it might play a role in modulating catalysis. The tail may allow CNPase to assume a specific orientation with respect to the myelin membrane and to act as a spacer between actin and microtubules when bound to a membrane.

### 2.5. Juxtanodin

Juxtanodin (Jux; also called ermin) is a monomeric 280-residue oligodendrocytic IDP localized to the juxtaparanodes of adaxonal non-compact myelin [163,164,165]. Jux is involved in the morphological regulation of oligodendrocytes, more specifically in the formation of arborizations [164,165,166]. Lowered Jux expression levels have been detected in epileptic patients [166].

**Figure 6:**
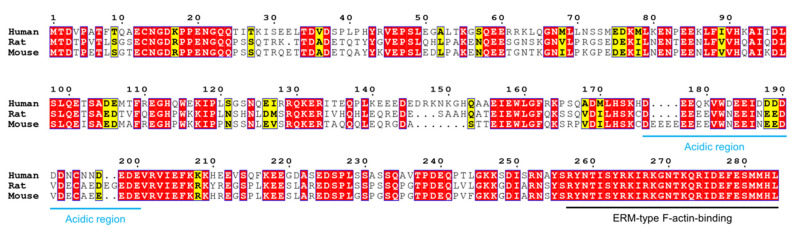
Comparison of human, rat, and mouse Jux. Jux is less conserved than MBP and MOBP, human Jux sharing only ~60% homology to mouse and rat Jux. An acidic region (blue) separates the protein into N- and C-terminal halves, the latter of which contains the fully conserved ERM-type actin binding domain (black). Residue numbering corresponds to human Jux.

Jux associates with the cytoskeleton, and the association is negatively regulated through phosphorylation [167]. The interaction involves filamentous actin, and it has been shown to be solely dependent on the C-terminal ezrin/radixin/moesin (ERM)-type F-actin-binding domain, which is the only region of Jux to share any homology with other proteins [163,167]. This domain is comprised of 30 amino acids, being the most conserved region in Jux (Figure 6); the last 14 residues are crucial for the actin interaction [163,167]. Data on the folding of this short segment are lacking, but disorder and secondary structure predictions suggest helical folding [163]. The phosphorylation target that regulates actin binding resides in this region as well [167]. Notably, Jux apparently does not affect the organization of actin filaments and only weakly inhibits their growth [163], which suggests that other factors are likely to be involved *in vivo* that allow Jux to regulate oligodendrocytic arborization. The inhibition is abolished by removal of the ERM domain, or by removal of the N-terminal half of Jux [163]. A prominent effect of Jux was recently observed in Jux-transfected retinal pigment epithelial cells, whereby the expression of Jux reorganized the actin cytoskeleton in a manner that affected cell morphology and size [168].

At the sequence level, Jux is not as conserved as MBP or MOBP, as e.g. human and rodent Jux share only ~60% identity (Figure 6). Nevertheless, in addition to the actin-binding domain, some conserved stretches are present. Of the ~280-residue sequence, just over 100 residues are charged, with 60% being acidic and 40% basic. An acidic region (residues 176-200 in human Jux) divides Jux into two halves: the N-terminal half is acidic, whereas the C-terminal half is basic. The significance of the central acidic region is unknown, as is the function of the N-terminal half. The C-terminal half of the sequence is more conserved between species, probably relating to its microfilament-binding function.

SAXS analysis revealed that Jux has several conformational populations in solution, which could be an indication of dynamic transient folding [163]. The entire intact protein was required for the conformational sampling, which implies long-range intramolecular interactions [163], possibly through the opposite net charges of the N- and C-terminal halves. Regions that associate with each other and (partially) fold in the process could therefore exist in Jux. This kind of conformational sampling is known to occur in some MoRFs, dubbed pre-formed structural elements [169], and could indicate binding sites for other interaction partners, hypothetically making Jux a cytoskeletal assembler protein. The only known binding partner for Jux is the actin filament, although Jux has been shown to co-localize with CNPase and to affect its trafficking [136,165]. Jux could be a bridging unit of cytoskeletal elements, as CNPase is known to bind microtubules and to anchor them to the plasma membrane [156,157,163]. Future studies include mapping of the interactome of Jux, in addition to structural studies on Jux bound to microfilaments.

### 2.6. Myelin-Associated Glycoprotein

**Figure 7:**
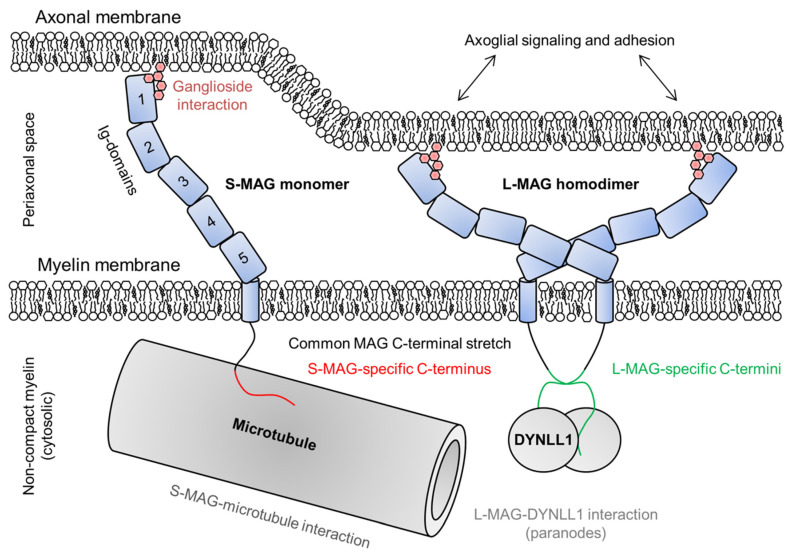
The roles of S- and L-MAG on both sides of the adaxonal layer. MAG is able to dimerize through Ig-domains 4 and 5, which determines the dimensions of MAG and thus the intermembrane distance within the periaxonal space [179]. MAG interacts with gangliosides on the axonal membrane and mediates bidirectional axoglial signaling [180], maintaining the width of the periaxonal space. In the cytosol beneath the adaxonal membrane, S-MAG interacts with microtubules and L-MAG with DYNLL1 [178,181].

Myelin-associated glycoprotein (MAG [170]) is a protein expressed in both the CNS and PNS at 1% and 0.3% of total protein, respectively [31,171]. MAG is produced as two alternatively spliced isoforms, L- and S-MAG. Both isoforms are type I transmembrane proteins with five glycosylated extracellular Ig-like domains, followed by a single transmembrane domain and a cytoplasmic extension. The cytoplasmic tails have 37 residues in common, ending in an isoform-specific C terminus (Figure 7) [172]. In the CNS, S-MAG and L-MAG can be detected at different stages of myelin development, L-MAG being present already during oligodendrocyte differentiation and myelination [173,174], whereas S-MAG mostly appears after myelin has formed [175]. The amounts of L- and S-MAG are roughly equal in mature CNS myelin [173]. In the PNS, S-MAG dominates in quantity, and the deletion of L-MAG in mice does not result in PNS demyelination [172,176]. In the CNS, S-MAG localizes mainly into the paranodal region of myelin, but in the PNS, it localizes much more diversely into the SLIs, paranodal loops, the adaxonal membrane, as well as ring-like accumulations around the myelin sheath in the abaxonal and adaxonal membranes [177]. L-MAG is practically absent in the adult PNS, but in the CNS, it is abundant and localizes mostly to the adaxonal layer along the internode and at the paranodes [177,178].

The five extracellular Ig-like domains of MAG span the periaxonal space and adhere to the axonal membrane via ganglioside interactions [179,180]. The Ig-like domains have been structurally characterized, and are thought to be rigid, defining the width of the periaxonal space [179]. MAG is involved in bidirectional axoglial signaling that regulates the thickness of axons as well as the expression and phosphorylation of axonal cytoskeletal proteins [180,182,183]. Ig-domains 4 and 5 mediate MAG dimerization (Figure 7) [179].

Both cytoplasmic extensions of MAG interact with Fyn tyrosine kinase, which is absolutely needed for the initiation of normal myelination [180]. The cytoplasmic domain of S-MAG is intrinsically disordered and interacts with Zn^2+^ and microtubules, indicating a structural role in non-compact myelin (Figure 7) [181,184]. The presence of the cytoplasmic extension of L-MAG is a prerequisite for CNS myelination [176]. Related to intracellular signaling, L-MAG has been shown to interact with cytosolic S100β and phospholipase Cγ [185,186]. The L-MAG cytoplasmic extension is intrinsically disordered and contains a β-MoRF that forms a complex with dynein light chain 1 (DYNLL1) in a 2:2 heterotetrameric assembly (Figure 7) [178]. DYNLL1 is known to dimerize disordered interaction partners [187], and L-MAG cytoplasmic domain dimerization induced by DYNLL1 may be mediated to the extracellular side of the membrane, affecting cell adhesion. The interaction site is non-canonical, lacking the consensus sequence found in other disordered DYNLL1-interacting proteins [178,188], and the binding site in L-MAG is conserved in mammals and reptiles [178]. A conserved Tyr in the direct vicinity of the binding site might have a regulatory role via phosphorylation [178,185]. Isoform-specific, DYNLL1-mediated dimerization of L-MAG could lead to different conformations or oligomeric states of the MAG extracellular domain and affect its affinity/avidity towards neuronal ligands.

### 2.7. Periaxin

Periaxin (PRX) is the most abundant non-compact myelin protein of the PNS, making up 16% of total protein mass [31]. PRX has two experimentally verified alternatively spliced isoforms: short (S-PRX) and long (L-PRX) [189]. The PRX isoforms share an N-terminal PDZ (PSD95/DLG1/ZO-1 [190]) domain of ~100 residues, which forms a structurally unique intertwined dimer [191]. The structure of the PDZ domain distinguishes PRX from all known PDZ domain-containing proteins [191,192], except the giant AHNAK2 nucleoprotein – the only protein with any sequence homology to PRX [191].

**Figure 8:**
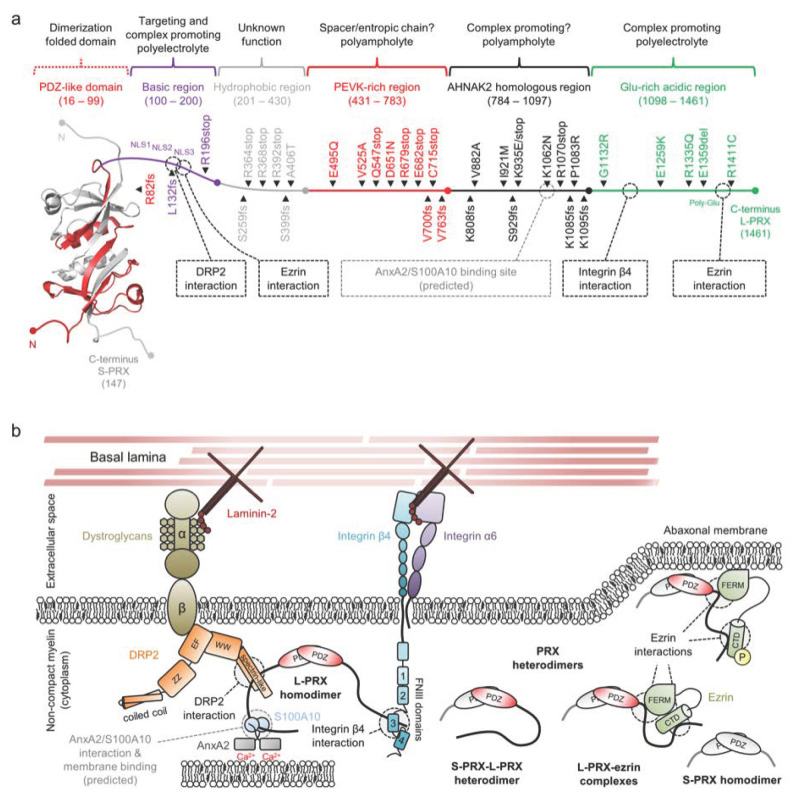
The structure and interactions of periaxin. (**a**) A schematic of a PRX heterodimer, with S-PRX in gray and L-PRX colored based on region, with the PDZ domain in red. L-PRX, apart from the PDZ-like domain, is predicted to be disordered [45], and can be divided into separate regions based on sequence composition. Peripheral neuropathy mutations are indicated alongside L-PRX. Dashed boxes and lines denote protein–protein interactions. L-PRX contains a predicted AnxA2 and S100A10 binding region, as reported earlier for AHNAK [195,196]. See Table 1 for mutation details. (**b**) L-PRX is an assembler within abaxonal non-compact myelin, linking dystroglycans and integrins together in membrane appositions, forming the periaxinosome. These interaction partners connect the Schwann cell basal lamina to the Schwann cell cytoplasm. S-PRX forms heterodimers with L-PRX, which might allow regulation of the cytoplasmic assembly as well as the nuclear export of L-PRX. Ezrin in complex with hetero- or homodimeric L-PRX might have relevance in such regulations, especially considering its phosphoregulated membrane-binding activity [197]. The function of the S-PRX homodimer is unknown. The significance of the putative L-PRX/AnxA2/Sl00A10 ternary complex could involve linking the entire assembly via AnxA2 and Ca^2+^ to the underlying membrane, possibly forming a structural basis for membrane appositions that line Cajal bands in myelinating Schwann cells.

The PDZ domain mediates both hetero- and homodimerization of S- and L-PRX [191,193]. In addition to the PDZ domain, S-PRX contains a C-terminal ~50-amino-acid tail of unknown function. For L-PRX, the C-terminal tail is 1300 amino acids [189]. This entire segment is predicted to be disordered [45], and based on its amino acid composition, the tail can be divided into five disordered regions: a strongly basic region (spanning amino acids 100–200 in human L-PRX), a hydrophobic region (201–430), a PEVK-rich region (431–783), a mildly basic region that shares homology with AHNAK2 (784–1097), and an acidic C-terminal region (1098–1461). The domain structure of an S- and L-PRX heterodimer is illustrated in Figure 8a. Refer to Supplementary Table S1 for a sequence analysis of L-PRX [194].

The basic region following the PDZ domain consists of a 100-residue Arg and Lys-rich polyelectrolytic sequence (Figure 8a,b). A tripartite nuclear localization signal (denoted NLS1, NLS2, and NLS3) resides at the N-terminal third of this region [198]. While this region marks L-PRX for nuclear trafficking [199], the same region mediates the interaction between L-PRX and the dystrophin-related protein 2 (DRP2)/dystroglycan complex [13]. The DRP2/dystroglycan complex is a major transmembrane assembly involved in the formation and stability of membrane appositions and Cajal bands in the Schwann cell abaxonal layer [13,200,201], and it is strictly found in appositions, whereas most of L-PRX is present in Cajal bands [13]. The interaction is thought to be mainly mediated by the DRP2 spectrin repeat domain, with possible involvement of the adjacent WW domain and NLS2/NLS3 in L-PRX (Figure 8b) [13,200]. Another recent discovery was the binding of the NLS3 region to the N-terminal FERM domain of ezrin, a member of the ERM-family of proteins that link cytoskeletal elements to membranes [197].

The basic region is followed by a hydrophobic region of unknown significance. This region is abundant in Ala, Leu, Pro, and Val, and the regional grand average of hydropathicity (GRAVY [202]: +0.202) is high compared to other regions in L-PRX (GRAVY values between −0.456 and −0.227).

The polyampholytic PEVK-rich region follows the hydrophobic region and is rich in Pro, Glu, Val, and Lys (Figure 8b). Such an amino acid composition is present in the giant elastic protein titin, where PEVK repeats form an extended entropic chain that contributes to the re-extension of the sarcomere after contraction, forming the basis of muscle relaxation [203]. In fact, the PEVK-rich region can be considered to be spanned by consecutive repeats of the pentapeptide motif [AGLMV]-[CEPQRS]-[DEKL]-[AILMPV]-[AEHKPQR] with very few gaps. A simpler curated motif of [KR][AGLV]P[DE]X (X = any residue) is very abundant. While no experimental evidence exists, the sequence composition of the L-PRX PEVK-rich region suggests at least partial extension/disorder that remains non-foldable under most conditions. A curious observation based on the mapping of PTMs in rat and mouse L-PRX reveals that the PEVK-rich region is devoid of phosphorylation sites, whereas other regions of L-PRX are subject to phosphorylation [131,204]. This might indicate the need to keep the region permanently extended, rather than the extension being regulated post-translationally over time. The relatively high content of evenly spaced Pro residues also suggests a potential hub for protein–protein interactions e.g., with SH3 domains.

**Figure 9:**
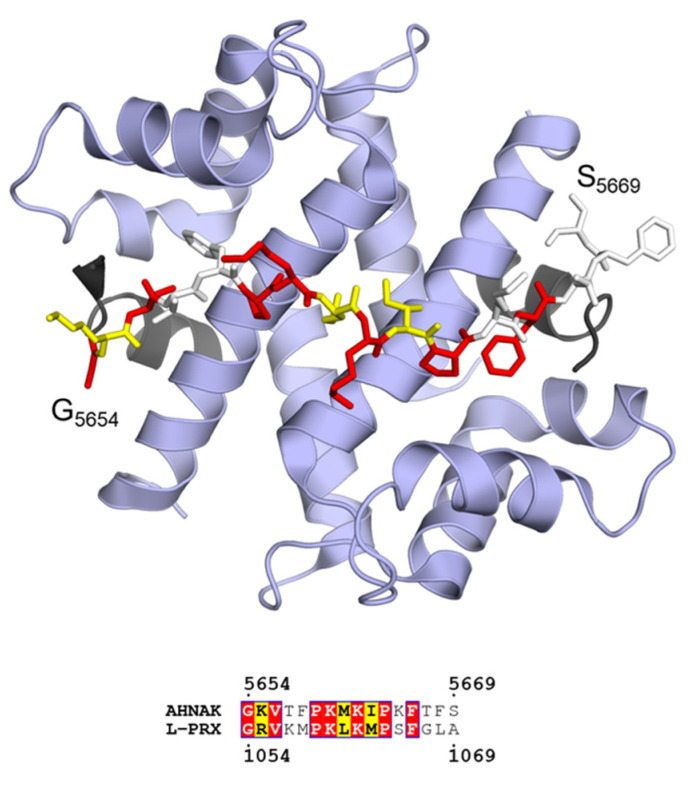
Ternary complex between AHNAK peptide (sticks), S100A10 (blue ribbon), and the acetylated N terminus of AnxA2 (black ribbon) in a 1:2:2 stoichiometry (PDB ID 4ftg [196]; top). Sequence alignment of the binding motif of AHNAK with L-PRX predicts a similar binding site in L-PRX (bottom). Coloring of the bound AHNAK peptide corresponds to residue conservation between AHNAK and L-PRX as evident from the sequence alignment.

The AHNAK2 homology region is polyampholytic, and basic residues slightly dominate over acidic ones (Figure 8b). Due to the presence of hydrophobic residues, the region should not be classified as a polar tract. The function of this region is currently unknown, but it shares a short region in common with AHNAK that has a potential binding partner. This region in AHNAK is an ι-MoRF that forms a ternary complex with the heterotetrameric assembly of annexin A2 (AnxA2) and S100A10 (also known as P11) in a 1:2:2 stoichiometry (Figure 9) [195,196]. The S100A10 dimer binds AHNAK and two acetylated N termini of AnxA2, and the structure suggests that the Ca^2+^-regulated AnxA2 can carry out its functions as part of the complex *e.g*. in membrane association [205]. AnxA2 is abundant in the PNS, where it mostly localizes to the cytosol of various cell types, including the non-compact myelin of Schwann cells [206]. S100A10 is also present in Schwann cells, where it interacts with AHNAK [207].

While L-PRX is predicted to be almost fully disordered, the acidic region is the only segment of L-PRX experimentally shown to be disordered: SAXS and CD experiments on a protein construct corresponding to the acidic region of rat L-PRX revealed that the protein is highly flexible in solution. In the same study, an interaction between the acidic region and the 3^rd^ fibronectin type III (FNIII-3) of integrin β4 was described (Figure 8a–b) [208]. The complex formation specifically involves integrin β4 and not integrin α6. Nevertheless, the accurate binding site within L-PRX remains elusive; while the integrin β4 FNIII-3 domain in isolation forms a stable complex with the acidic region, L-PRX-derived peptides corresponding to various conserved linear stretches within the acidic region did not bind to FNIII-3 [208]. The binding could be mediated by several weakly binding short linear motifs that together result in a stable complex, rather than the binding involving a single MoRF. It is possible that the PRX acidic region forms a fuzzy complex with FNIII-3. Observing the structure of FNIII-3, it is plausible that L-PRX binds through a β-MoRF that is stabilized by adjacent β-strands in FNIII-3. In the C-terminal end of the acidic domain, another ezrin binding site has been reported. While the basic domain interacts with the FERM domain of ezrin, the acidic domain binds to the ezrin C-terminal domain. Both domain interactions occur simultaneously, and interestingly, the binding of ezrin to the acidic domain can occur synergistically with integrin β4 binding [197].

The different regions of L-PRX most likely function together to achieve its function. L-PRX mainly populates the outermost cytosolic compartment of myelinating Schwann cells, the abaxonal layer, where it contributes to the formation of membrane appositions and the stability of Cajal bands [13,200,201]. The localization of PRX depends on O-linked *N*-acetylglucosamination [209]. When correctly localized, L-PRX assembles abundant structural membrane protein complexes together, bridging the extracellular basal lamina with Schwann cell cytoplasmic components [13,201,208]. We call this continuous, macroscopic protein meshwork the periaxinosome. In its homodimeric state, L-PRX can potentially form large supramolecular complexes, in which the laminin-bound DRP2-dystroglycan complex and the integrin α6β4 complex are adjoined and correctly spaced by the PEVK-rich domain. These two complexes are crucial for achieving correctly matured PNS myelin [210,211,212]. If the L-PRX/AnxA2/S100A10 complex exists, the periaxinosome assembly could be Ca^2+^-bridged via AnxA2 to the abaxonal membrane or even an apposing membrane underneath, which could be speculated to form a molecular basis for membrane appositions in Schwann cells (Figure 8b). Another possibility for forming membrane appositions would be the association of phosphorylated ezrin with an apposing membrane via its FERM domain. In this scenario, ezrin should still be able to remain bound to L-PRX via its C-terminal domain, although L-PRX-ezrin complexes were suggested to partake in regulating the assembly of the periaxinosome rather than being structural parts of it (see below) [197].

As a potential regulatory mechanism, heterodimerization between S- and L-PRX has been proposed to dissociate large clustered complexes when needed, and as a regulatory mechanism of the nuclear export of L-PRX (Figure 8b) [193]. Indeed, as S-PRX lacks all NLS motifs, it is predominantly found in the cytoplasm, although it localizes near the outer rim of the nucleus in mouse cerebral endothelial cells [213]. Such localization in Schwann cells remains to be verified. 

## 3. Selected Examples of IDPs in Demyelination

Demyelination arises from the destruction of the myelin sheath, which slows down rapid saltatory conduction and in some cases results in axonal degeneration. Demyelination can arise from mutations in myelin protein genes [214], from mitochondrial abnormalities [215], from the induction of the unfolded protein response [216], or from environmental factors, such as viral infections or medication [217,218]. In this chapter, we will specifically focus on the involvement of MBP in MS, as well as the role of PRX in peripheral neuropathies.

### 3.1. Basic Proteins and Multiple Sclerosis

MS is the best-known and most common demyelinating condition of the CNS [219]. In MS, myelin is destroyed in an autoimmune attack either by activated T-cells that have crossed the blood brain barrier [220,221], or by microglia, the immune cells of the CNS [222]. Various antigens are linked to MS, many of which originate from myelin proteins, such as CNPase [223], MAG [224], MBP [93,225,226], MOBP [227,228], or PLP [229], or from peptides of viral origin [225,230,231].

The antigenic epitopes of MBP and MOBP are known [93,225,226,227,228], and both reside in highly conserved regions (see Figure 3; Figure 4). In the case of MBP, the antigenic region has been structurally characterized in complex with a major histocompatibility complex class 2 protein, and it assumes an extended conformation in the bound state [106,232,233,234,235]. Molecular mimicry has been proposed as a mechanism for MBP-borne MS: the release of the antigenic epitope in the form of (auto)proteolytic peptides that resemble viral peptides can be detected by the immune system, which results in an attack against myelin and subsequent demyelination [23,225,230,236,237,238]. While free disordered MBP is likely to be susceptible to degradation [226,239,240,241], the recently described intermediate state in MBP-mediated membrane stacking could equally well be a target for proteolysis, especially since membrane stacking is dependent on the concentration of available MBP [24]. The presence of acidic lipids has been shown to accelerate the digestion of MBP by cathepsin D [242]. In addition, the proteolytic susceptibility of deiminated MBP is higher [226,239].

**Figure 10:**
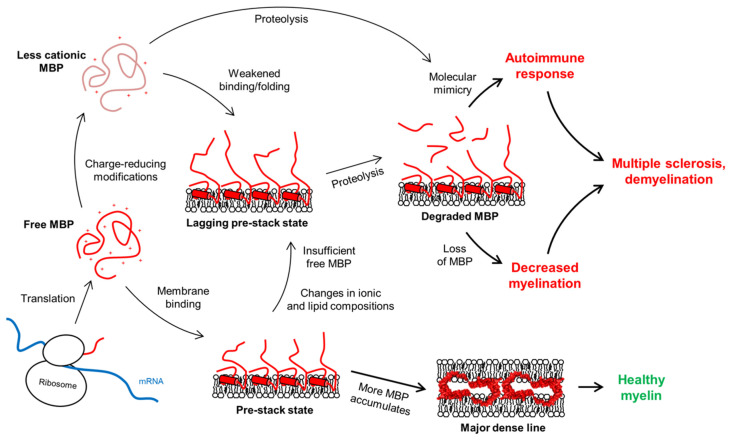
The fate of the MDL formation is governed by the concentration of active MBP (red), which normally would associate with membranes, form a pre-stack state, and continue to accumulate until stable membrane stacks form. MS is linked to an autoimmune response and loss of myelin, which could stem from molecular mimicry. The immune system recognizes antigenic MBP peptides formed via proteolysis of free or membrane-bound MBP. Changes in membrane lipid composition or concentration of intracellular ions could pre-expose to this process [53,90]. Changes in the PTMs of MBP have additionally been shown to play a role [60,226,239], as might lowered MBP expression levels [24].

In MS, the lipid composition and ion content of myelin are altered, affecting the membrane-stacking activity of MBP [243,244]. Ca^2+^ is a major divalent cation in myelin and has numerous roles in oligodendrocyte differentiation and myelination [128,245,246,247,248,249]. Ca^2+^ has been shown to affect the production of MBP [250], and more notably, its membrane binding [53,90,244]. On the other hand, MBP affects the amount of Ca^2+^ in oligodendrocytes [72,251]. Additionally, Zn^2+^ is an abundant trace element in myelin and has been found to interact with MBP and boost its ability to bridge membranes in compact myelin [86,87,88,89,252,253]. Zn^2+^ has also been connected to demyelination, notably MS [129,130]. Taking into account all these factors, one can speculate that pro-pathological changes in the myelin environment, as well as changes in PTMs in the MBP pool, could influence the membrane-stacking activity of MBP, increasing proteolytic susceptibility. This is a plausible pathway that involves molecular mimicry and, combined with the subsequent loss of MBP and decreased myelination [254], a pathological mechanism that could contribute to MS (Figure 10).

### 3.2. Periaxin and Peripheral Neuropathies

Peripheral neuropathies are a diverse group of conditions of the peripheral nervous system, all of which share in common a significant deceleration of nerve impulse conduction. They arise from either demyelination or axonal degeneration. The most common peripheral neuropathy is Charcot–Marie–Tooth disease (CMT), with all its subtypes collectively affecting 2.8 million people worldwide. The symptomatic spectrum of CMT is broad, including tingling sensation and numbness of the limbs, weakness, fatigue, pain, muscle spasms, loss of muscle mass, and the very common hallmark feature of the disease: arched feet. The onset of CMT is generally broad, ranging from early childhood to ~40 years [255].

Mutations in PRX result in CMT type 4F (CMT4F) and Dejerine–Sottas syndrome (DSS), which are severe demyelinating forms of CMT with morphological changes in Schwann cells (Figure 8a, Table 1). For example, Cajal bands are abolished and the abaxonal layer appears uniform with the R1070stop mutation, which removes the entire acidic region [208]. Similarly, deletion mutations in DRP2 that abolish the interaction with L-PRX are detrimental to myelin and result in disease [201,256]. The effects of mutations that introduce a premature stop codon or a frameshift are easily explained by loss of function, due to large truncations in L-PRX. Unfortunately, most point mutations in L-PRX remain uncharacterized at the protein level. The regions with the most missense mutations could be involved in as-of-yet undiscovered interactions. On the other hand, mutations could induce folding of disordered regions, protein aggregation, or problems with protein synthesis. Some mutations can be linked to potential abolishment of protein–protein interactions, such as K1062N, which lies in the middle of the predicted AnxA2/S100A10 binding site in the AHNAK2 homology region [257].

## 4. Future Research Directions

Despite large efforts to uncover the mysteries of IDPs in myelin, the work is not over yet. MBP is an example of how decades of work have allowed us to understand how the multifunctionality of an IDP is linked to disorder-to-order transitions [65]. Still, new results may generate more questions than answers. The discovery of the pre-stack state in MBP-driven myelin compaction raises new ideas regarding MS etiology. Is it possible that a disordered protein brush undergoes degradation, and could this release autoantigenic epitopes? The involvement of various splice isoforms of MBP, PTMs, divalent cations, and specific lipid species should be re-addressed. Additionally, structural aspects of MBP functions apart from membrane stacking are subjects of future research: disorder-to-order transitions in cytoskeletal interactions, for instance, would not only help us understand the effects of PTMs and the isoform composition of the heterogeneous MBP pool, but also to use MBP as a prototype to elucidate general attributes of IDPs in other systems.

Biophysical methodologies will continue to be highly important in the study of MBP and IDPs in general. CD and NMR spectroscopy are powerful tools in directly probing disorder [275,276]. Small-angle scattering methods are sensitive to aggregation, but useful in probing conformational sampling in solution [277], complementing for example Förster resonance energy transfer experiments and computational methods [278,279]. Techniques that require isotope labeling, such as neutron methods and NMR spectroscopy, face their own challenges during protein production. However, folding experiments involving partially labeled proteins could prove to be highly useful. As demonstrated using reflectometry and neutron scattering, the conformations of proteins in solution can be probed under membrane-mimicking conditions [24], and introducing a second level of contrast with partially labeled proteins could allow further dissection of molecular binding modes. The individually folding segments of MBP have been studied with NMR spectroscopy, but for instance intein coupling could enable specific labeling of segments of interest in full-length MBP for NMR and neutron studies [280,281]. This would allow mapping of, for example, the segment of MBP inserting first into membranes, when the pre-stack state forms.

What is the relevance of MOBP in myelin and MS? Is Zn^2+^ important for its membrane binding? These are two fundamental questions that need to be addressed in the future. The high conservation of MOBP suggests an important role in myelin. At the same time, Zn^2+^ is abundant in myelin [127], and while it can contribute to bridging membranes together by itself or through protein–cation interactions [89,184,252,282], it most likely populates binding sites in zinc fingers [283], like the one predicted in MOBP [48].

Jux binds actin filaments, but the molecular details are poorly understood [163]. Jux is not as conserved as other IDPs in myelin, but small conserved segments are present that could be involved in other functions (Figure 6). An important future goal is to understand the entire interactome of Jux. Systematic approaches towards uncovering binding partners would help in placing Jux into a functional context.

The potential regulatory role of S- and L-PRX heterodimerization needs to be characterized at the molecular level, in order to shed light on PRX nuclear trafficking and the formation of the periaxinosome. Are PNS myelin membrane appositions driven by high local concentrations of DRP2 that recruit PRX, or does PRX recruit DRP2 and cluster it to form appositions? Could ezrin be similarly involved in the recruitment of L-PRX for integrin β4 binding, as hinted by their synergistic interaction? The involvement of AnxA2 and S100A10 is speculative, and the putative formation of a ternary complex with L-PRX should be studied. The role of the PEVK-rich domain as a molecular spacer is intriguing. In membrane appositions, the bridge between the abaxonal membrane and the membrane underneath is currently not known, but L-PRX could fill in this spot.

## 5. Conclusions

A clear divide exists between the IDPs of compact and non-compact myelin. Compact myelin IDPs are highly basic and conserved, implying functional importance of membrane binding and eventual stacking with concurrent partial folding of the IDP. In non-compact myelin, disorder is relevant for protein–protein interactions and the formation of large protein complexes that may link the myelinating cell cytoskeleton to extracellular components. Both myelin compartments are likely to involve IDP-related molecular phase separation and the formation of membraneless organelles. While the mysteries of myelin protein structure–function relationships are slowly unraveling, we are still a long way from understanding the basic molecular essence of myelin, which is one of the most unique biological compartments in vertebrates. Multidisciplinary approaches coupled with hybrid structural biology methodologies will enable a deeper insight into the molecular interplay in myelination and related neurological disease.

## Figures and Tables

**Table 1:** PRX mutations, related neuropathies and potential molecular mechanisms.

Mutation ^1^	Neuropathy	(Potential) Molecular Impact	Reference(s)
R82fs	DSS	Tail loss; loss of interactions	[258]
L132fs	CMT4F	Tail loss; loss of interactions	[259]
R196stop	CMT4F		[260]
S259fs	CMT4F	Loss of hydrophobic, PEVK-rich, AHNAK2 homology and acidic regions; loss of interactions	[261]
R364stop	CMT4F	Loss of PEVK-rich, AHNAK2 homology and acidic regions; loss of interactions	[262]
R368stop	DSS	Loss of PEVK-rich, AHNAK2 homology and acidic regions; loss of interactions	[263]
R392stop	DSS	Loss of PEVK-rich, AHNAK2 homology and acidic regions; loss of interactions	[264]
S399fs	CMT4F	Loss of PEVK-rich, AHNAK2 homology and acidic regions; loss of interactions	[265]
A406T	DSS		[263]
E495Q	DSS		[263]
V525A	CMT4F		[260,266]
Q547stop	CMT4F	Loss of PEVK-rich (partial), AHNAK2 homology and acidic regions; loss of interactions	[261]
D651N	CMT4F		[267]
R679stop	DSS	Loss of PEVK-rich (partial), AHNAK2 homology and acidic regions; loss of interactions	[264]
E682stop	CMT4F	Loss of PEVK-rich (partial), AHNAK2 homology and acidic regions; loss of interactions	[261]
A700fs	CMT4F		[268]
C715stop	DSS	Loss of PEVK-rich (partial), AHNAK2 homology and acidic regions; loss of interactions	[258]
V763fs	DSS	Loss of PEVK-rich (partial), AHNAK2 homology and acidic regions; loss of interactions	[263]
K808fs	CMT4F	Loss of AHNAK2 homology and acidic regions; loss of interactions	[261]
V882A	DSS		[263,269]
I921M	DSS		[263]
S929fs	DSS	Loss of AHNAK2 homology and acidic regions; loss of interactions	[263]
K935E	DSS		[263]
K935stop	DSS	Loss of acidic domain; loss of integrin interaction	[263]
K1062N	CMT4F	(Loss of predicted AnxA2/S100A10 interaction?)	[257]
R1070stop	CMT4F	Loss of acidic domain; loss of integrin interaction	[208,259,267,270,271,272]
P1083R	DSS		[265]
E1085fs	CMT4F	Loss of acidic domain; loss of integrin interaction	[273]
K1095fs	CMT4F	Loss of acidic domain; loss of integrin interaction	[274]
G1132R	DSS		[263]
E1259K	DSS		[263]
R1335Q ^2^	CMT		[266]
E1359del	DSS		[263]
R1411C	DSS		[263]

^1^ fs denotes frame shift mutation, stop denotes nonsense mutation. ^2^ Found together with V525A in a complex neuropathy associated with dysarthria, hypermobile joints, and cerebellar symptoms.

## Supplementary Materials

The following are available online at https://www.mdpi.com/2073-4409/9/2/470/s1, Table S1: Sequence analysis of all human L-PRX regions (UniProtKB - Q9BXM0-1) using ProtParam.

## References

[1] Wang S.S.H., Shultz J.R., Burish M.J., Harrison K.H., Hof P.R., Towns L.C., Wagers M.W., Wyatt K.D. (2008). Shaping of white matter composition by biophysical scaling constraints. J. Neurosci..

[2] Lundgaard I., Luzhynskaya A., Stockley J.H., Wang Z., Evans K.A., Swire M., Volbracht K., Gautier H.O.B., Franklin R.J.M., ffrench-Constant C. (2013). Neuregulin and BDNF Induce a Switch to NMDA Receptor-Dependent Myelination by Oligodendrocytes. PLoS. Biol..

[3] Salzer J.L. (2015). Schwann Cell Myelination. Cold Spring Harbor Perspect. Biol..

[4] Nave K. (2010). Myelination and the trophic support of long axons. Nat. Rev. Neurosci..

[5] Young J.Z. (1938). The functioning of the giant nerve fibres of the squid. J. Exp. Biol..

[6] Simons M., Trotter J. (2007). Wrapping it up: The cell biology of myelination. Curr. Opin. Neurobiol..

[7] Zuchero J.B., Fu M., Sloan S.A., Ibrahim A., Olson A., Zaremba A., Dugas J.C., Wienbar S., Caprariello A.V., Kantor C. (2015). CNS myelin wrapping is driven by actin disassembly. Dev. Cell.

[8] Hartline D.K. (2008). What is myelin?. Neuron Glia Biol..

[9] Fünfschilling U., Supplie L.M., Mahad D., Boretius S., Saab A.S., Edgar J., Brinkmann B.G., Kassmann C.M., Tzvetanova I.D., Möbius W. (2012). Glycolytic oligodendrocytes maintain myelin and long-term axonal integrity. Nature.

[10] Court F.A., Wrabetz L., Feltri M.L. (2006). Basal lamina: Schwann cells wrap to the rhythm of space-time. Curr. Opin. Neurobiol..

[11] Court F., Sherman D., Pratt T., Garry E., Ribchester R., Cottrell D., Fleetwood-Walker S., Brophy P. (2004). Restricted growth of Schwann cells lacking Cajal bands slows conduction in myelinated nerves. Nature.

[12] Court F.A., Hewitt J.E., Davies K., Patton B.L., Uncini A., Wrabetz L., Feltri M.L. (2009). A Laminin-2, Dystroglycan, Utrophin Axis Is Required for Compartmentalization and Elongation of Myelin Segments. J. Neurosci..

[13] Sherman D.L., Wu L.M.N., Grove M., Gillespie C.S., Brophy P.J. (2012). Drp2 and Periaxin Form Cajal Bands with Dystroglycan But Have Distinct Roles in Schwann Cell Growth. J. Neurosci..

[14] Micu I., Plemel J.R., Caprariello A.V., Nave K., Stys P.K. (2018). Axo-myelinic neurotransmission: A novel mode of cell signalling in the central nervous system. Nat. Rev. Neurosci..

[15] Saher G., Brugger B., Lappe-Siefke C., Möbius W., Tozawa R., Wehr M., Wieland F., Ishibashi S., Nave K. (2005). High cholesterol level is essential for myelin membrane growth. Nat. Neurosci..

[16] Saher G., Quintes S., Nave K. (2011). Cholesterol: A novel regulatory role in myelin formation. Neuroscientist.

[17] Aggarwal S., Yurlova L., Simons M. (2011). Central nervous system myelin: Structure, synthesis and assembly. Trends Cell Biol..

[18] Jo E., Boggs J. (1995). Aggregation of Acidic Lipid Vesicles by Myelin Basic-Protein–Dependence on Potassium Concentration. Biochemistry.

[19] Luo X., Sharma D., Inouye H., Lee D., Avila R.L., Salmona M., Kirschner D.A. (2007). Cytoplasmic domain of human myelin protein zero likely folded as beta-structure in compact myelin. Biophys. J..

[20] Suresh S., Wang C., Nanekar R., Kursula P., Edwardson J.M. (2010). Myelin basic protein and myelin protein 2 act synergistically to cause stacking of lipid bilayers. Biochemistry.

[21] Wang C., Neugebauer U., Bürck J., Myllykoski M., Baumgärtel P., Popp J., Kursula P. (2011). Charge isomers of myelin basic protein: Structure and interactions with membranes, nucleotide analogues, and calmodulin. PLoS ONE.

[22] Ruskamo S., Yadav R.P., Sharma S., Lehtimäki M., Laulumaa S., Aggarwal S., Simons M., Bürck J., Ulrich A.S., Juffer A.H. (2014). Atomic resolution view into the structure-function relationships of the human myelin peripheral membrane protein P2. Acta Cryst. D.

[23] Tuusa J., Raasakka A., Ruskamo S., Kursula P. (2017). Myelin-derived and putative molecular mimic peptides share structural properties in aqueous and membrane-like environments. Mult. Scler. Demyelinating Disord..

[24] Raasakka A., Ruskamo S., Kowal J., Barker R., Baumann A., Martel A., Tuusa J., Myllykoski M., Bürck J., Ulrich A.S. (2017). Membrane Association Landscape of Myelin Basic Protein Portrays Formation of the Myelin Major Dense Line. Sci. Rep..

[25] Schmitt F.O., Bear R.S., Clark G.L. (1935). The Role of Lipoids in the X-Ray Diffraction Patterns of Nerve. Science.

[26] Schmitt F.O., Bear R.S., Palmer K.J. (1941). X-ray diffraction studies on the structure of the nerve myelin sheath. J. Cell. Physiol..

[27] Robertson J.D., Nachmansohn D. (1960). The Molecular Biology of Cell Membranes.

[28] Robertson J.D. (1987). The Early Days of Electron-Microscopy of Nerve-Tissue and Membranes. Int. Rev. Cytol..

[29] Meller K. (1990). Cryoelectron Microscopy of Vitrified Nerve Myelin. Cell Tissue Res..

[30] Aggarwal S., Yurlova L., Snaidero N., Reetz C., Frey S., Zimmermann J., Paehler G., Janshoff A., Friedrichs J., Müller D.J. (2011). A Size Barrier Limits Protein Diffusion at the Cell Surface to Generate Lipid-Rich Myelin-Membrane Sheets. Dev. Cell.

[31] Patzig J., Jahn O., Tenzer S., Wichert S.P., de Monasterio-Schrader P., Rosfa S., Kuharev J., Yan K., Bormuth I., Bremer J. (2011). Quantitative and Integrative Proteome Analysis of Peripheral Nerve Myelin Identifies Novel Myelin Proteins and Candidate Neuropathy Loci. J. Neurosci..

[32] de Monasterio-Schrader P., Jahn O., Tenzer S., Wichert S.P., Patzig J., Werner H.B. (2012). Systematic approaches to central nervous system myelin. Cell. Mol. Life Sci..

[33] Yin X., Baek R., Kirschner D., Peterson A., Fujii Y., Nave K., Macklin W., Trapp B. (2006). Evolution of a neuroprotective function of central nervous system myelin. J. Cell Biol..

[34] Campi G., Di Gioacchino M., Poccia N., Ricci A., Burghammer M., Bianconi A. (2017). Intrinsic dynamical fluctuations of PNS myelin. arXiv.

[35] Shapiro L., Doyle J., Hensley P., Colman D., Hendrickson W. (1996). Crystal structure of the extracellular domain from P-0, the major structural protein of peripheral nerve myelin. Neuron.

[36] Inouye H., Tsuruta H., Sedzik J., Uyemura K., Kirschner D.A. (1999). Tetrameric Assembly of Full-Sequence Protein Zero Myelin Glycoprotein by Synchrotron X-Ray Scattering. Biophys. J..

[37] Thompson A.J., Cronin M.S., Kirschner D.A. (2002). Myelin protein zero exists as dimers and tetramers in native membranes of Xenopus laevis peripheral nerve. J. Neurosci. Res..

[38] Favereaux A., Lagueny A., Vital A., Schmitter J., Chaignepain S., Ferrer X., Labatut-Cazabat I., Vital C., Petry K. (2003). Serum IgG antibodies to P0 dimer and 35 kDa P0 related protein in neuropathy associated with monoclonal gammopathy. J. Neurol. Neurosurg. Psychiatry.

[39] Plotkowski M.L., Kim S., Phillips M.L., Partridge A.W., Deber C.M., Bowie J.U. (2007). Transmembrane domain of myelin protein zero can form dimers: Possible implications for myelin construction. Biochemistry.

[40] Toyama B., Savas J., Park S., Harris M., Ingolia N., Yates J., Hetzer M. (2013). Identification of Long-Lived Proteins Reveals Exceptional Stability of Essential Cellular Structures. Cell.

[41] Boggs J.M. (2006). Myelin basic protein: A multifunctional protein. Cell Mol. Life Sci..

[42] Harauz G., Ladizhansky V., Boggs J.M. (2009). Structural Polymorphism and Multifunctionality of Myelin Basic Protein. Biochemistry.

[43] Fulton D., Paez P.M., Campagnoni A.T. (2010). The multiple roles of myelin protein genes during the development of the oligodendrocyte. ASN Neuro.

[44] Raasakka A., Kursula P. (2014). The myelin membrane-associated enzyme 2’,3’-cyclic nucleotide 3’-phosphodiesterase: On a highway to structure and function. Neurosci. Bull..

[45] Han H., Myllykoski M., Ruskamo S., Wang C., Kursula P. (2013). Myelin-specific proteins: A structurally diverse group of membrane-interacting molecules. Biofactors.

[46] Arroyo E., Scherer S. (2000). On the molecular architecture of myelinated fibers. Histochem. Cell Biol..

[47] Kursula P. (2001). The current status of structural studies on proteins of the myelin sheath (review). Int. J. Mol. Med..

[48] Kursula P. (2008). Structural properties of proteins specific to the myelin sheath. Amino Acids.

[49] Orthmann-Murphy J.L., Abrams C.K., Scherer S.S. (2008). Gap junctions couple astrocytes and oligodendrocytes. J. Mol. Neurosci..

[50] Liang X., Gomez G.A., Yap A.S. (2015). Current perspectives on cadherin-cytoskeleton interactions and dynamics. Cell Health Cytoskelet..

[51] Luo X., Inouye H., Gross A.A.R., Hidalgo M.M., Sharma D., Lee D., Avila R.L., Salmona M., Kirschner D.A. (2007). Cytoplasmic domain of zebrafish myelin protein zero: Adhesive role depends on beta-conformation. Biophys. J..

[52] Raasakka A., Ruskamo S., Kowal J., Han H., Baumann A., Myllykoski M., Fasano A., Rossano R., Riccio P., Bürck J. (2019). Molecular structure and function of myelin protein P0 in membrane stacking. Sci. Rep..

[53] Raasakka A., Jones N., Hoffmann S.V., Kursula P. (2019). Ionic strength and calcium regulate the membrane interactions of myelin basic protein and the cytoplasmic domain of myelin protein zero. Biochem. Biophys. Res. Commun..

[54] Raasakka A., Ruskamo S., Barker R., Krokengen O.C., Vatne G.H., Kristiansen C.K., Hallin E.I., Skoda M.W.A., Bergmann U., Wacklin-Knecht H. (2019). Neuropathy-related mutations alter the membrane binding properties of the human myelin protein P0 cytoplasmic tail. PLoS ONE.

[55] Myllykoski M., Baumgärtel P., Kursula P. (2012). Conformations of peptides derived from myelin-specific proteins in membrane-mimetic conditions probed by synchrotron radiation CD spectroscopy. Amino Acids.

[56] van der Lee R., Buljan M., Lang B., Weatheritt R.J., Daughdrill G.W., Dunker A.K., Fuxreiter M., Gough J., Gsponer J., Jones D.T. (2014). Classification of Intrinsically Disordered Regions and Proteins. Chem. Rev..

[57] Sedzik J., Kirschner D.A. (1992). Is myelin basic protein crystallizable?. Neurochem Res..

[58] Pedraza L. (1997). Nuclear transport of myelin basic protein. J. Neurosci. Res..

[59] Moscarello M.A., Juurlink B.H.J., Devon R.M., Doucette J.R., Nazarali A.J., Schreyer D.J., Verge V.M.K. (1997). Myelin Basic Protein, the “Executive” Molecule of the Myelin Membrane.

[60] Kim J., Mastronardi F., Wood D., Lubman D., Zand R., Moscarello M. (2003). Multiple sclerosis–An important role for post-translational modifications of myelin basic protein in pathogenesis. Mol. Cell. Proteom..

[61] Harauz G., Ishiyama N., Hill C., Bates I., Libich D., Fares C. (2004). Myelin basic protein–diverse conformational states of an intrinsically unstructured protein and its roles in myelin assembly and multiple sclerosis. Micron.

[62] Harauz G., Musse A.A. (2007). A tale of two citrullines–Structural and functional aspects of myelin basic protein deimination in health and disease. Neurochem. Res..

[63] Baron W., Hoekstra D. (2010). On the biogenesis of myelin membranes: Sorting, trafficking and cell polarity. FEBS Lett..

[64] Müller C., Bauer N.M., Schaefer I., White R. (2013). Making myelin basic protein–from mRNA transport to localized translation. Front. Cell. Neurosci..

[65] Vassall K.A., Bamm V.V., Harauz G. (2015). MyelStones: The executive roles of myelin basic protein in myelin assembly and destabilization in multiple sclerosis. Biochem. J..

[66] Robert X., Gouet P. (2014). Deciphering key features in protein structures with the new ENDscript server. Nucleic Acids Res..

[67] Ridsdale R., Beniac D., Tompkins T., Moscarello M., Harauz G. (1997). Three-dimensional structure of myelin basic protein II. Molecular modeling and considerations of predicted structures in multiple sclerosis. J. Biol. Chem..

[68] Pribyl T.M., Campagnoni C.W., Kampf K., Kashima T., Handley V.W., McMahon J., Campagnoni A.T. (1993). The Human Myelin Basic-Protein Gene is Included within a 179-Kilobase Transcription Unit–Expression in the Immune and Central Nervous Systems. Proc. Natl. Acad. Sci. USA.

[69] Campagnoni A.T., Pribyl T.M., Campagnoni C.W., Kampf K., Amurumarjee S., Landry C.F., Handley V.W., Newman S.L., Garbay B., Kitamura K. (1993). Structure and Developmental Regulation of Golli-Mbp, a 105-Kilobase Gene that Encompasses the Myelin Basic-Protein Gene and is Expressed in Cells in the Oligodenrocyte Lineage in the Brain. J. Biol. Chem..

[70] Mendz G.L., Barden J.A., Martenson R.E. (1995). Conformation of a Tetradecapeptide Epitope of Myelin Basic-Protein. Eur. J. Biochem..

[71] Feng J., Hu Y.K., Xie L., Colwell C.S., Shao X.M., Sun X., Chen B., Tang H., Campagnoni A.T. (2006). Golli protein negatively regulates store depletion-induced calcium influx in T cells. Immunity.

[72] Smith G.S., Paez P.M., Spreuer V., Campagnoni C.W., Boggs J.M., Campagnoni A.T., Harauz G. (2011). Classical 18.5-and 21.5-kDa isoforms of myelin basic protein inhibit calcium influx into oligodendroglial cells, in contrast to golli isoforms. J. Neurosci. Res..

[73] Li Z., Zhang Y., Li D., Feng Y. (2000). Destabilization and mislocalization of myelin basic protein mRNAs in quaking dysmyelination lacking the QKI RNA-binding proteins. J. Neurosci..

[74] Torvund-Jensen J., Steengaard J., Reimer L., Fihl L.B., Laursen L.S. (2014). Transport and translation of MBP mRNA is regulated differently by distinct hnRNP proteins. J. Cell. Sci..

[75] Fernandes A., Campagnoni C., Kampf K., Feng J., Handley V., Schonmann V., Bongarzone E., Reyes S., Campagnoni A. (2004). Identification of a protein that interacts with the Golli-Myelin basic protein and with nuclear-LIM interactor in the nervous system. J. Neurosci. Res..

[76] Smith G.S.T., Seymour L.V., Boggs J.M., Harauz G. (2012). The 21.5-kDa isoform of myelin basic protein has a non-traditional PY-nuclear-localization signal. Biochem. Biophys. Res. Commun..

[77] Smith G.S.T., Samborska B., Hawley S.P., Klaiman J.M., Gillis T.E., Jones N., Boggs J.M., Harauz G. (2013). Nucleus-localized 21.5-kDa myelin basic protein promotes oligodendrocyte proliferation and enhances neurite outgrowth in coculture, unlike the plasma membrane-associated 18.5-kDa isoform. J. Neurosci. Res..

[78] Smith G.S.T., Homchaudhuri L., Boggs J.M., Harauz G. (2012). Classic 18.5-and 21.5-kDa myelin basic protein isoforms associate with cytoskeletal and SH3-domain proteins in the immortalized N19-oligodendroglial cell line stimulated by phorbol ester and IGF-1. Neurochem. Res..

[79] Smith G.S.T., De Avila M., Paez P.M., Spreuer V., Wills M.K.B., Jones N., Boggs J.M., Harauz G. (2012). Proline substitutions and threonine pseudophosphorylation of the SH3 ligand of 18.5-kDa myelin basic protein decrease its affinity for the Fyn-SH3 domain and alter process development and protein localization in oligodendrocytes. J. Neurosci. Res..

[80] De Avila M., Vassall K.A., Smith G.S.T., Bamm V.V., Harauz G. (2014). The proline-rich region of 18.5 kDa myelin basic protein binds to the SH3-domain of Fyn tyrosine kinase with the aid of an upstream segment to form a dynamic complex in vitro. Biosci. Rep..

[81] Boggs J.M., Homchaudhuri L., Ranagaraj G., Liu Y., Smith G.S.T., Harauz G. (2014). Interaction of myelin basic protein with cytoskeletal and signaling proteins in cultured primary oligodendrocytes and N19 oligodendroglial cells. BMC Res. Notes.

[82] Robb N.D., Chen W.H. (1990). Myelin Basic Protein Interaction with Calmodulin and Gangliosides. J. Neurosci. Res..

[83] Harauz G., Ishiyama N., Bates I. (2000). Analogous standard motifs in myelin basic protein and in MARCKS. Mol. Cell. Biochem..

[84] Libich D., Hill C., Haines J., Harauz G. (2003). Myelin basic protein has multiple calmodulin-binding sites. Biochem. Biophys. Res. Commun..

[85] Bamm V.V., De Avila M., Smith G.S.T., Ahmed M.A.M., Harauz G. (2011). Structured Functional Domains of Myelin Basic Protein: Cross Talk between Actin Polymerization and Ca2+-Dependent Calmodulin Interaction. Biophys. J..

[86] Cavatorta P., Giovanelli S., Bobba A., Riccio P., Szabo A.G., Quagliariello E. (1994). Myelin Basic-Protein Interaction with Zinc and Phosphate–Fluorescence Studies on the Water-Soluble Form of the Protein. Biophys. J..

[87] Smith G.S.T., Chen L., Bamm V.V., Dutcher J.R., Harauz G. (2010). The interaction of zinc with membrane-associated 18.5 kDa myelin basic protein: An attenuated total reflectance-Fourier transform infrared spectroscopic study. Amino Acids.

[88] Baran C., Smith G.S.T., Bamm V.V., Harauz G., Lee J.S. (2010). Divalent cations induce a compaction of intrinsically disordered myelin basic protein. Biochem. Biophys. Res. Commun..

[89] Earl C., Chantry A., Mohammad N., Glynn P. (1988). Zinc Ions Stabilize the Association of Basic-Protein with Brain Myelin Membranes. J. Neurochem..

[90] Nawaz S., Kippert A., Saab A.S., Werner H.B., Lang T., Nave K., Simons M. (2009). Phosphatidylinositol 4,5-Bisphosphate-Dependent Interaction of Myelin Basic Protein with the Plasma Membrane in Oligodendroglial Cells and Its Rapid Perturbation by Elevated Calcium. J. Neurosci..

[91] Zhang C., Walker A.K., Zand R., Moscarello M.A., Yan J.M., Andrews P.C. (2012). Myelin Basic Protein Undergoes a Broader Range of Modifications in Mammals than in Lower Vertebrates. J. Proteome Res..

[92] Wood D.D., Moscarello M.A. (1989). The Isolation, Characterization, and Lipid-Aggregating Properties of a Citrulline Containing Myelin Basic-Protein. J. Biol. Chem..

[93] Wood D., Bilbao J., OConnors P., Moscarello M. (1996). Acute multiple sclerosis (Marburg type) is associated with developmentally immature myelin basic protein. Ann. Neurol..

[94] Turner R.S., Chou C.H.J., Mazzei G.J., Dembure P., Kuo J.F. (1984). Phospholipid-Sensitive Ca2+-Dependent Protein-Kinase Preferentially Phosphorylates Serine-115 of Bovine Myelin Basic-Protein. J. Neurochem..

[95] Kishimoto A., Nishiyama K., Nakanishi H., Uratsuji Y., Nomura H., Takeyama Y., Nishizuka Y. (1985). Studies on the Phosphorylation of Myelin Basic-Protein by Protein-Kinase C and Adenosine 3’-5’-Monophosphate-Dependent Protein-Kinase. J. Biol. Chem..

[96] Schulz P., Cruz T.F., Moscarello M.A. (1988). Endogenous Phosphorylation of Basic-Protein in Myelin of Varying Degrees of Compaction. Biochemistry.

[97] Erickson A.K., Payne D.M., Martino P.A., Rossomando A.J., Shabanowitz J., Weber M.J., Hunt D.F., Sturgill T.W. (1990). Identification by Mass-Spectrometry of Threonine-97 in Bovine Myelin Basic-Protein as a Specific Phosphorylation Site for Mitogen-Activated Protein-Kinase. J. Biol. Chem..

[98] Ramwani J., Moscarello M.A. (1990). Phosphorylation of Charge Isomers (Components) of Human Myelin Basic-Protein–Identification of Phosphorylated Sites. J. Neurochem..

[99] Wang Q.M., Smith J.B., Harrison M.L., Geahlen R.L. (1991). Identification of Tyrosine-67 in Bovine Brain Myelin Basic-Protein as a Specific Phosphorylation Site for Thymus-P56lck. Biochem. Biophys. Res. Commun..

[100] Zand R., Li M., Jin X., Lubman D. (1998). Determination of the sites of posttranslational modifications in the charge isomers of bovine myelin basic protein by capillary electrophoresis mass spectroscopy. Biochemistry.

[101] Wood D.D., Ackerley C.A., van den Brand B., Zhang L., Raijmakers R., Mastronardi F.G., Moscarello M.A. (2008). Myelin localization of peptidylarginine deiminases 2 and 4: Comparison of PAD2 and PAD4 activities. Lab. Invest..

[102] Beniac D.R., Wood D.D., Palaniyar N., Ottensmeyer P., Moscarello M.A., Harauz G. (1999). Marburg’s variant of multiple sclerosis correlates with a less compact structure of myelin basic protein. Mol. Cell. Biol. Res. Commun..

[103] McLaurin J., Ackerley C.A., Moscarello M.A. (1993). Localization of Basic-Proteins in Human Myelin. J. Neurosci. Res..

[104] Boggs J.M., Rangaraj G., Hill C.M.D., Bates I.R., Heng Y.M., Harauz G. (2005). Effect of arginine loss in myelin basic protein, as occurs in its deiminated charge isoform, on mediation of actin polymerization and actin binding to a lipid membrane in vitro. Biochemistry.

[105] Tompkins T.A., Moscarello M.A. (1993). Stimulation of Bovine Brain Phospholipase-C Activity by Myelin Basic-Protein Requires Arginyl Residues in Peptide Linkage. Arch. Biochem. Biophys..

[106] Li Y., Li H., Martin R., Mariuzza R. (2000). Structural basis for the binding of an immunodominant peptide from myelin basic protein in different registers by two HLA-DR2 proteins. J. Mol. Biol..

[107] Bates I., Feix J., Boggs J., Harauz G. (2004). An immunodominant epitope of myelin basic protein is an amphipathic alpha-helix. J. Biol. Chem..

[108] Hu Y., Doudevski I., Wood D., Moscarello M., Husted C., Genain C., Zasadzinski J., Israelachvili J. (2004). Synergistic interactions of lipids and myelin basic protein. Proc. Natl. Acad. Sci. USA.

[109] Musse A.A., Gao W., Rangaraj G., Boggs J.M., Harauz G. (2009). Myelin basic protein co-distributes with other PI(4,5)P-2-sequestering proteins in Triton X-100 detergent-resistant membrane microdomains. Neurosci. Lett..

[110] Lee D.W., Banquy X., Kristiansen K., Kaufman Y., Boggs J.M., Israelachvili J.N. (2014). Lipid domains control myelin basic protein adsorption and membrane interactions between model myelin lipid bilayers. Proc. Natl. Acad. Sci. USA.

[111] Widder K., Träger J., Kerth A., Harauz G., Hinderberger D. (2018). Interaction of Myelin Basic Protein with Myelin-like Lipid Monolayers at Air-Water Interface. Langmuir.

[112] Widder K., Harauz G., Hinderberger D. (2020). Myelin basic protein (MBP) charge variants show different sphingomyelin-mediated interactions with myelin-like lipid monolayers. Biochim. Biophys. Acta Biomembr..

[113] Ishiyama N., Bates I., Hill C., Wood D., Matharu P., Viner N., Moscarello M., Harauz G. (2001). The effects of deimination of myelin basic protein on structures formed by its interaction with phosphoinositide-containing lipid monolayers. J. Struct. Biol..

[114] Aggarwal S., Snaidero N., Paehler G., Frey S., Sanchez P., Zweckstetter M., Janshoff A., Schneider A., Weil M., Schaap I.A.T. (2013). Myelin membrane assembly is driven by a phase transition of myelin basic proteins into a cohesive protein meshwork. PLoS Biol..

[115] Beniac D., Luckevich M., Czarnota G., Tompkins T., Ridsdale R., Ottensmeyer F., Moscarello M., Harauz G. (1997). Three-dimensional structure of myelin basic protein 1. Reconstruction via angular reconstitution of randomly oriented single particles. J. Biol. Chem..

[116] Haas H., Oliveira C., Torriani I., Polverini E., Fasano A., Carlone G., Cavatorta P., Riccio P. (2004). Small angle x-ray scattering from lipid-bound myelin basic protein in solution. Biophys. J..

[117] Libich D.S., Harauz G. (2008). Backbone dynamics of the 18.5 kDa isoform of myelin basic protein reveals transient alpha-helices and a calmodulin-binding site. Biophys. J..

[118] Ahmed M.A.M., De Avila M., Polverini E., Bessonov K., Bamm V.V., Harauz G. (2012). Solution nuclear magnetic resonance structure and molecular dynamics simulations of a murine 18.5 kDa myelin basic protein segment (S72-S107) in association with dodecylphosphocholine micelles. Biochemistry.

[119] Yin X., Peterson J., Gravel M., Braun P., Trapp B. (1997). CNP overexpression induces aberrant oligodendrocyte membranes and inhibits MBP accumulation and myelin compaction. J. Neurosci. Res..

[120] Snaidero N., Velte C., Myllykoski M., Raasakka A., Ignatev A., Werner H.B., Erwig M.S., Möbius W., Kursula P., Nave K. (2017). Antagonistic functions of MBP and CNP establish cytosolic channels in CNS myelin. Cell Rep..

[121] Yamamoto Y., Mizuno R., Nishimura T., Ogawa Y., Yoshikawa H., Fujimura H., Adachi E., Kishimoto T., Yanagihara T., Sakoda S. (1994). Cloning and Expression of Myelin-Associated Oligodendrocytic Basic-Protein–a Novel Basic-Protein Constituting the Central-Nervous-System Myelin. J. Biol. Chem..

[122] Holz A., Schaeren-Wiemers N., Schaefer C., Pott U., Colello R., Schwab M. (1996). Molecular and developmental characterization of novel cDNAs of the myelin-associated oligodendrocytic basic protein. J. Neurosci..

[123] Montague P., McCallion A., Davies R., Griffith I. (2006). Myelin-Associated Oligodendrocytic Basic Protein: A Family of Abundant CNS Myelin Proteins in Search of a Function. Dev. Neurosci..

[124] Kosaras B., Kirschner D.A. (1990). Radial Component of CNS Myelin–Junctional Subunit Structure and Supramolecular Assembly. J. Neurocytol..

[125] Yamamoto Y., Yoshikawa H., Nagano S., Kondoh G., Sadahiro S., Gotow T., Yanagihara T., Sakoda S. (1999). Myelin-associated oligodendrocytic basic protein is essential for normal arrangement of the radial component in central nervous system myelin. Eur. J. Neurosci..

[126] Yoshikawa H. (2001). Myelin-associated oligodendrocytic basic protein modulates the arrangement of radial growth of the axon and the radial component of myelin. Med. Electron Microsc..

[127] Bourre J.M., Cloez I., Galliot M., Buisine A., Dumont O., Piciotti M., Prouillet F., Bourdon R. (1987). Occurrence of manganese, copper and zinc in myelin. Alterations in the peripheral nervous system of dysmyelinating trembler mutant are at variance with brain mutants (quaking and shiverer). Neurochem. Int..

[128] Stys P.K., Lehning E., Saubermann A.J., LoPachin R.M. (1997). Intracellular Concentrations of Major Ions in Rat Myelinated Axons and Glia: Calculations Based on Electron Probe X-Ray Microanalyses. J. Neurochem..

[129] Bonaventura P., Benedetti G., Albarède F., Miossec P. (2015). Zinc and its role in immunity and inflammation. Autoimmun. Rev..

[130] Choi B.Y., Jung J.W., Suh S.W. (2017). The Emerging Role of Zinc in the Pathogenesis of Multiple Sclerosis. Int. J. Mol. Sci..

[131] Lundby A., Secher A., Lage K., Nordsborg N.B., Dmytriyev A., Lundby C., Olsen J.V. (2012). Quantitative maps of protein phosphorylation sites across 14 different rat organs and tissues. Nat. Commun..

[132] Cilia E., Pancsa R., Tompa P., Lenaerts T., Vranken W.F. (2014). The DynaMine webserver: Predicting protein dynamics from sequence. Nucleic Acids Res..

[133] PONDR^®^ Predictor of Natural Disordered Regions. http://www.pondr.com.

[134] Kelley L.A., Mezulis S., Yates C.M., Wass M.N., Sternberg M.J.E. (2015). The Phyre2 web portal for protein modeling, prediction and analysis. Nat. Protoc..

[135] Schäfer I., Müller C., Luhmann H.J., White R. (2016). MOBP levels are regulated by Fyn kinase and affect the morphological differentiation of oligodendrocytes. J. Cell. Sci..

[136] Trapp B.D., Bernier L., Andrews S.B., Colman D.R. (1988). Cellular and subcellular distribution of 2’,3’-cyclic nucleotide 3’-phosphodiesterase and its mRNA in the rat central nervous system. J. Neurochem..

[137] Radtke C., Sasaki M., Lankford K.L., Gallo V., Kocsis J.D. (2011). CNPase Expression in Olfactory Ensheathing Cells. J. Biomed. Biotechnol..

[138] O’Neill R., Minuk J., Cox M., Braun P., Gravel M. (1997). CNP2 mRNA directs synthesis of both CNP1 and CNP2 polypeptides. J. Neurosci. Res..

[139] McFerran B., Burgoyne R. (1997). 2’,3’-Cyclic nucleotide 3’-phosphodiesterase is associated with mitochondria in diverse adrenal cell types. J. Cell. Sci..

[140] Lee J., O’Neill R., Park M., Gravel M., Braun P. (2006). Mitochondrial localization of CNP2 is regulated by phosphorylation of the N-terminal targeting signal by PKC: Implications of a mitochondrial function for CNP2 in glial and non-glial cells. Mol. Cell. Neurosci..

[141] Myllykoski M., Seidel L., Muruganandam G., Raasakka A., Torda A.E., Kursula P. (2016). Structural and functional evolution of 2’,3’-cyclic nucleotide 3’-phosphodiesterase. Brain Res..

[142] Myllykoski M., Kursula P. (2010). Expression, purification, and initial characterization of different domains of recombinant mouse 2’,3’-cyclic nucleotide 3’-phosphodiesterase, an enigmatic enzyme from the myelin sheath. BMC Res. Notes..

[143] Myllykoski M., Itoh K., Kangas S., Heape A., Kang S., Lubeck G., Kursula I., Kursula P. (2012). The N-terminal domain of the myelin enzyme 2’,3’-cyclic nucleotide 3’-phosphodiesterase: Direct molecular interaction with the calcium sensor calmodulin. J. Neurochem..

[144] Stingo S., Masullo M., Polverini E., Laezza C., Ruggiero I., Arcone R., Ruozi E., Dal Piaz F., Malfitano A.M., D’Ursi A.M. (2007). The N-terminal domain of 2’,3’-cyclic nucleotide 3’-phosphodiesterase harbors a GTP/ATP binding site. Chem. Biol. Drug Des..

[145] Drummond G.I., Iyer N.T., Keith J. (1962). Hydrolysis of ribonucleoside 2′,3′-cyclic phosphates by a diesterase from brain. J. Biol. Chem..

[146] Kozlov G., Lee J., Elias D., Gravel M., Gutierrez P., Ekiel I., Braun P., Gehring K. (2003). Structural evidence that brain cyclic nucleotide phosphodiesterase is a member of the 2H phosphodiesterase superfamily. J. Biol. Chem..

[147] Sakamoto Y., Tanaka N., Ichimiya T., Kurihara T., Nakamura K. (2005). Crystal structure of the catalytic fragment of human brain 2’,3’-cyclic-nucleotide 3’-phosphodiesterase. J. Mol. Biol..

[148] Myllykoski M., Raasakka A., Han H., Kursula P. (2012). Myelin 2’,3’-Cyclic Nucleotide 3’-Phosphodiesterase: Active-Site Ligand Binding and Molecular Conformation. PLoS ONE.

[149] Myllykoski M., Raasakka A., Lehtimäki M., Han H., Kursula I., Kursula P. (2013). Crystallographic Analysis of the Reaction Cycle of 2’,3’-Cyclic Nucleotide 3’-Phosphodiesterase, a Unique Member of the 2H Phosphoesterase Family. J. Mol. Biol..

[150] Raasakka A., Myllykoski M., Laulumaa S., Lehtimäki M., Härtlein M., Moulin M., Kursula I., Kursula P. (2015). Determinants of ligand binding and catalytic activity in the myelin enzyme 2’,3’-cyclic nucleotide 3’-phosphodiesterase. Sci. Rep..

[151] Lee J., Gravel M., Gao E., O’Neill R., Braun P. (2001). Identification of essential residues in 2’,3’-cyclic nucleotide 3’-phosphodiesterase–Chemical modification and site-directed mutagenesis to investigate the role of cysteine and histidine residues in enzymatic activity. J. Biol. Chem..

[152] Verrier J., Jackson T., Bansal R., Kochanek P.M., Jackson E. (2012). Oligodendrocyte 2’,3’-Cyclic Nucleotide 3’-Phosphodiesterase Participates in Localized Adenosine Production: Possible Role in Traumatic Brain Injury. J. Neurotrauma.

[153] Verrier J.D., Jackson T.C., Bansal R., Kochanek P.M., Puccio A.M., Okonkwo D.O., Jackson E.K. (2012). The brain in vivo expresses the 2’,3’-cAMP-adenosine pathway. J. Neurochem..

[154] Verrier J.D., Jackson T.C., Gillespie D.G., Janesko-Feldman K., Bansal R., Goebbels S., Nave K., Kochanek P.M., Jackson E.K. (2013). Role of CNPase in the oligodendrocytic extracellular 2’,3’-cAMP-adenosine pathway. Glia.

[155] Gravel M., Robert F., Kottis V., Gallouzi I., Pelletier J., Braun P.E. (2009). 2’,3’-Cyclic Nucleotide 3’-Phosphodiesterase: A Novel RNA-Binding Protein That Inhibits Protein Synthesis. J. Neurosci. Res..

[156] Bifulco M., Laezza C., Stingo S., Wolff J. (2002). 2’,3’-Cyclic nucleotide 3’-phosphodiesterase: A membrane-bound, microtubule-associated protein and membrane anchor for tubulin. Proc. Natl. Acad. Sci. USA.

[157] Lee J., Gravel M., Zhang R., Thibault P., Braun P. (2005). Process outgrowth in oligodendrocytes is mediated by CNP, a novel microtubule assembly myelin protein. J. Cell Biol..

[158] De Angelis D.A., Braun P.E. (1996). 2’,3’-Cyclic Nucleotide 3’-Phosphodiesterase Binds to Actin-Based Cytoskeletal Elements in an Isoprenylation-Independent Manner. J. Neurochem..

[159] Azarashvili T., Krestinina O., Galvita A., Grachev D., Baburina Y., Stricker R., Evtodienko Y., Reiser G. (2009). Ca2+-dependent permeability transition regulation in rat brain mitochondria by 2’,3’-cyclic nucleotides and 2’,3’-cyclic nucleotide 3’-phosphodiesterase. Am. J. Physiol. Cell Physiol..

[160] Baburina Y., Azarashvili T., Grachev D., Krestinina O., Galvita A., Stricker R., Reiser G. (2015). Mitochondrial 2’,3’-cyclic nucleotide 3’-phosphodiesterase (CNP) interacts with mPTP modulators and functional complexes (I-V) coupled with release of apoptotic factors. Neurochem. Int..

[161] Baburina Y., Odinokova I., Azarashvili T., Akatov V., Sotnikova L., Krestinina O. (2018). Possible Involvement of 2’,3’-Cyclic Nucleotide-3’-Phosphodiesterase in the Protein Phosphorylation-Mediated Regulation of the Permeability Transition Pore. Int. J. Mol. Sci..

[162] Esposito C., Scrima M., Carotenuto A., Tedeschi A., Rovero P., D’Errico G., Malfitano A.M., Bifulco M., D’Ursi A.M. (2008). Structures and micelle locations of the nonlipidated and lipidated C-terminal membrane anchor of 2’,3’-cyclic nucleotide-3’-phosphodiesterase. Biochemistry.

[163] Ruskamo S., Chukhlieb M., Vahokoski J., Bhargav S.P., Liang F., Kursula I., Kursula P. (2012). Juxtanodin is an intrinsically disordered F-actin-binding protein. Sci. Rep..

[164] Brockschnieder D., Sabanay H., Riethmacher D., Peles E. (2006). Ermin, a myelinating oligodendrocyte-specific protein that regulates cell morphology. J. Neurosci..

[165] Zhang B., Cao Q., Guo A., Chu H., Chan Y., Buschdorf J., Low B., Ling E., Liang F. (2005). Juxtanodin: An oligodendroglial protein that promotes cellular arborization and 2’,3’-cyclic nucleotide-3’-phosphodiesterase trafficking. Proc. Natl. Acad. Sci. USA.

[166] Wang T., Jia L., Lu B., Liu B., Wang W., Wang F., Yang G., Bu X., Yao L., Zhang B. (2011). Human Ermin (hErmin), a new oligodendrocyte-specific cytoskeletal protein related to epileptic seizure. Brain Res..

[167] Meng J., Xia W., Tang J., Tang B.L., Liang F. (2010). Dephosphorylation-dependent Inhibitory Activity of Juxtanodin on Filamentous Actin Disassembly. J. Biol. Chem..

[168] Liang F., Hwang J.H., Tang N.W., Hunziker W. (2018). Juxtanodin in retinal pigment epithelial cells: Expression and biological activities in regulating cell morphology and actin cytoskeleton organization. J. Comp. Neurol..

[169] Fuxreiter M., Simon I., Friedrich P., Tompa P. (2004). Preformed structural elements feature in partner recognition by intrinsically unstructured proteins. J. Mol. Biol..

[170] Lehmann F., Gathje H., Kelm S., Dietz F. (2004). Evolution of sialic acid-binding proteins: Molecular cloning and expression of fish siglec-4. Glycobiology.

[171] Jahn O., Tenzer S., Werner H.B. (2009). Myelin Proteomics: Molecular Anatomy of an Insulating Sheath. Mol. Neurobiol..

[172] Miescher G., Lutzelschwab R., Erne B., Ferracin F., Huber S., Steck A. (1997). Reciprocal expression of myelin-associated glycoprotein splice variants in the adult human peripheral and central nervous systems. Mol. Brain Res..

[173] Butt A., Ibrahim M., Gregson N., Berry M. (1998). Differential expression of the L- and S-isoforms of myelin associated glycoprotein (MAG) in oligodendrocyte unit phenotypes in the adult rat anterior medullary velum. J. Neurocytol..

[174] Keita M., Magy L., Heape A., Richard L., Piaser M., Vallat J. (2002). Immunocytological studies of L-MAG expression regulation during myelination of embryonic brain cell cocultures. Dev. Neurosci..

[175] Ishiguro H., Sato S., Fujita N., Inuzuka T., Nakano R., Miyatake T. (1991). Immunohistochemical Localization of Myelin-Associated Glycoprotein Isoforms during the Development in the Mouse-Brain. Brain Res..

[176] Fujita N., Kemper A., Dupree J., Nakayasu H., Bartsch U., Schachner M., Maeda N., Suzuki K., Suzuki K., Popko B. (1998). The cytoplasmic domain of the large myelin-associated glycoprotein isoform is needed for proper CNS but not peripheral nervous system myelination. J. Neurosci..

[177] Erb M., Flueck B., Kern F., Erne B., Steck A., Schaeren-Wiemers N. (2006). Unraveling the differential expression of the two isoforms of myelin-associated glycoprotein in a mouse expressing GFP-tagged S-MAG specifically regulated and targeted into the different myelin compartments. Mol. Cell. Neurosci..

[178] Myllykoski M., Eichel M.A., Jung R.B., Kelm S., Werner H.B., Kursula P. (2018). High-affinity heterotetramer formation between the large myelin-associated glycoprotein and the dynein light chain DYNLL1. J. Neurochem..

[179] Pronker M.F., Lemstra S., Snijder J., Heck A.J.R., Thies-Weesie D.M.E., Pasterkamp R.J., Janssen B.J.C. (2016). Structural basis of myelin-associated glycoprotein adhesion and signalling. Nat. Commun..

[180] Quarles R.H. (2007). Myelin-associated glycoprotein (MAG): Past, present and beyond. J. Neurochem..

[181] Kursula P., Lehto V., Heape A. (2001). The small myelin-associated glycoprotein binds to tubulin and microtubules. Mol. Brain Res..

[182] Yin X., Crawford T., Griffin J., Tu P., Lee V., Li C., Roder J., Trapp B. (1998). Myelin-associated glycoprotein is a myelin signal that modulates the caliber of myelinated axons. J. Neurosci..

[183] Dashiell S., Tanner S., Pant H., Quarles R. (2002). Myelin-associated glycoprotein modulates expression and phosphorylation of neuronal cytoskeletal elements and their associated kinases. J. Neurochem..

[184] Kursula P., Meriläinen G., Lehto V., Heape A. (1999). The small myelin-associated glycoprotein is a zinc-binding protein. J. Neurochem..

[185] Jaramillo M.L., Afar D.E.H., Almazan G., Bell J.C. (1994). Identification of Tyrosine-620 as the Major Phosphorylation Site of Myelin-Associated Glycoprotein and its Implication in Interacting with Signaling Molecules. J. Biol. Chem..

[186] Kursula P., Tikkanen G., Lehto V., Nishikimi M., Heape A. (1999). Calcium-dependent interaction between the large myelin-associated glycoprotein and S100 beta. J. Neurochem..

[187] Barbar E. (2008). Dynein light chain LC8 is a dimerization hub essential in diverse protein networks. Biochemistry.

[188] Clark S., Nyarko A., Loehr F., Karplus P.A., Barbar E. (2016). The Anchored Flexibility Model in LC8 Motif Recognition: Insights from the Chica Complex. Biochemistry.

[189] Dytrych L., Sherman D., Gillespie C., Brophy P. (1998). Two PDZ domain proteins encoded by the murine periaxin gene are the result of alternative intron retention and are differentially targeted in Schwann cells. J. Biol. Chem..

[190] Kennedy M.B. (1995). Origin of Pdz (Dhr, Glgf) Domains. Trends Biochem. Sci..

[191] Han H., Kursula P. (2014). Periaxin and AHNAK Nucleoprotein 2 Form Intertwined Homodimers through Domain Swapping. J. Biol. Chem..

[192] Lee H., Zheng J.J. (2010). PDZ domains and their binding partners: Structure, specificity, and modification. Cell Commun. Signal..

[193] Yang Y., Shi Y. (2015). L-periaxin interacts with S-periaxin through its PDZ domain. Neurosci. Lett..

[194] Gasteiger E., Hoogland C., Gattiker A., Duvaud S., Wilkins M., Appel R., Bairoch A., Walker J. (2005). Protein Identification and Analysis Tools on the ExPASy Server.

[195] Dempsey B.R., Rezvanpour A., Lee T., Barber K.R., Junop M.S., Shaw G.S. (2012). Structure of an Asymmetric Ternary Protein Complex Provides Insight for Membrane Interaction. Structure.

[196] Ozorowski G., Milton S., Luecke H. (2013). Structure of a C-terminal AHNAK peptide in a 1:2:2 complex with S100A10 and an acetylated N-terminal peptide of annexin A2. Acta Cryst. D.

[197] Guo T., Zhang L., Xiao H., Yang Y., Shi Y. (2020). Ezrin interacts with L-periaxin by the “head to head and tail to tail” mode and influences the location of L-periaxin in Schwann cell RSC96. Biochim. Biophys. Acta Gen. Subj..

[198] Sherman D., Brophy P. (2000). A tripartite nuclear localization signal in the PDZ-domain protein L-periaxin. J. Biol. Chem..

[199] Shi Y., Zhang L., Yang T. (2014). Nuclear Export of L-Periaxin, Mediated by Its Nuclear Export Signal in the PDZ Domain. PLoS ONE.

[200] Sherman D., Fabrizi C., Gillespie C., Brophy P. (2001). Specific disruption of a Schwann cell dystrophin-related protein complex in a demyelinating neuropathy. Neuron.

[201] Brennan K.M., Bai Y., Pisciotta C., Wang S., Feely S.M.E., Hoegger M., Gutmann L., Moore S.A., Gonzalez M., Sherman D.L. (2015). Absence of Dystrophin Related Protein-2 disrupts Cajal bands in a patient with Charcot-Marie-Tooth disease. Neuromusc. Disord..

[202] Kyte J., Doolittle R.F. (1982). A Simple Method for Displaying the Hydropathic Character of a Protein. J. Mol. Biol..

[203] Forbes J.G., Jin A.J., Ma K., Gutierrez-Cruz G., Tsai W.L., Wang K. (2005). Titin PEVK segment: Charge-driven elasticity of the open and flexible polyampholyte. J. Muscle Res. Cell. Motil..

[204] Huttlin E.L., Jedrychowski M.P., Elias J.E., Goswami T., Rad R., Beausoleil S.A., Villen J., Haas W., Sowa M.E., Gygi S.P. (2010). A Tissue-Specific Atlas of Mouse Protein Phosphorylation and Expression. Cell.

[205] Gerke V., Moss S. (2002). Annexins: From structure to function. Physiol. Rev..

[206] Hayashi A., Nakashima K., Yamagishi K., Hoshi T., Suzuki A., Baba H. (2007). Localization of annexin II in the paranodal regions and Schmidt-Lanterman incisures in the peripheral nervous system. Glia.

[207] Donato R., Cannon B.R., Sorci G., Riuzzi F., Hsu K., Weber D.J., Geczy C.L. (2013). Functions of S100 Proteins. Curr. Mol. Med..

[208] Raasakka A., Linxweiler H., Brophy P.J., Sherman D.L., Kursula P. (2019). Direct binding of the flexible C-terminal segment of periaxin to β4 integrin suggests a molecular basis for CMT4F. Front. Mol. Neurosci..

[209] Kim S., Maynard J.C., Sasaki Y., Strickland A., Sherman D.L., Brophy P.J., Burlingame A.L., Milbrandt J. (2016). Schwann Cell O-GlcNAc Glycosylation Is Required for Myelin Maintenance and Axon Integrity. J. Neurosci..

[210] Einheber S., Milner T.A., Giancotti F., Salzer J.L. (1993). Axonal Regulation of Schwann-Cell Integrin Expression Suggests a Role for Alpha-6-Beta-4 in Myelination. J. Cell Biol..

[211] Masaki T., Matsumura K., Hirata A., Yamada H., Hase A., Arai K., Shimizu T., Yorifuji H., Motoyoshi K., Kamakura K. (2002). Expression of dystroglycan and the laminin-alpha 2 chain in the rat peripheral nerve during development. Exp. Neurol..

[212] Wang M.M., Zhang X., Lee S.J., Maripudi S., Keep R.F., Johnson A.M., Stamatovic S.M., Andjelkovic A.V. (2018). Expression of periaxin (PRX) specifically in the human cerebrovascular system: PDZ domain-mediated strengthening of endothelial barrier function. Sci. Rep..

[213] Pancsa R., Schad E., Tantos A., Tompa P. (2019). Emergent functions of proteins in non-stoichiometric supramolecular assemblies. Biochim. Biophys. Acta Proteins Proteom..

[214] Oksenberg J., Seboun E., Hauser S. (1996). Genetics of demyelinating diseases. Brain Pathol..

[215] Carvalho K.S. (2013). Mitochondrial Dysfunction in Demyelinating Diseases. Semin. Pediatr. Neurol..

[216] Lin W., Stone S. (2020). Unfolded protein response in myelin disorders. Neural Regen. Res..

[217] Torkildsen O., Brunborg L.A., Myhr K.M., Bø L. (2008). The cuprizone model for demyelination. Acta Neurol. Scand..

[218] Virtanen J.O., Jacobson S. (2012). Viruses and Multiple Sclerosis. CNS Neurol. Disord. Drug Targets.

[219] Browne P., Chandraratna D., Angood C., Tremlett H., Baker C., Taylor B.V., Thompson A.J. (2014). Atlas of Multiple Sclerosis 2013: A growing global problem with widespread inequity. Neurology.

[220] Namer I.J., Steibel J., Poulet P., Armspach J.P., Mohr M., Mauss Y., Chambron J. (1993). Blood-Brain-Barrier Breakdown in Mbp-Specific T-Cell Induced Experimental Allergic Encephalomyelitis–a Quantitative Invivo Mri Study. Brain.

[221] Galea I., Bernardes-Silva M., Forse P.A., van Rooijen N., Liblau R.S., Perry V.H. (2007). An antigen-specific pathway for CD8 T cells across the blood-brain barrier. J. Exp. Med..

[222] Luo C., Jian C., Liao Y., Huang Q., Wu Y., Liu X., Zou D., Wu Y. (2017). The role of microglia in multiple sclerosis. Neuropsychiatr. Dis. Treat..

[223] Muraro P., Kalbus M., Afshar G., McFarland H., Martin R. (2002). T cell response to 2’,3’-cyclic nucleotide 3’-phosphodiesterase (CNPase) in multiple sclerosis patients. J. Neuroimmunol..

[224] Andersson M., Yu M., Söderström M., Weerth S., Baig S., Linington C., Solders G., Link H. (2002). Multiple MAG peptides are recognized by circulating T and B lymphocytes in polyneuropathy and multiple sclerosis. Eur. J. Neurol..

[225] Tejada-Simon M., Zang Y., Hong J., Rivera V., Zhang J. (2003). Cross-reactivity with myelin basic protein and human herpesvirus-6 in multiple sclerosis. Ann. Neurol..

[226] Musse A., Boggs J., Harauz G. (2006). Deimination of membrane-bound myelin basic protein in multiple sclerosis exposes an immunodominant epitope. Proc. Natl. Acad. Sci. USA.

[227] Holz A., Bielekova B., Martin R., Oldstone M. (2000). Myelin-associated oligodendrocytic basic protein: Identification of an encephalitogenic epitope and association with multiple sclerosis. J. Immunol..

[228] Kaushansky N., Eisenstein M., Zilkha-Falb R., Ben-Nun A. (2010). The myelin-associated oligodendrocytic basic protein (MOBP) as a relevant primary target autoantigen in multiple sclerosis. Autoimmun. Rev..

[229] Kondo T., Yamamura T., Inobe J., Ohashi T., Takahashi K., Tabira T. (1996). TCR repertoire to proteolipid protein (PLP) in multiple sclerosis (MS): Homologies between PLP-specific T cells and MS-associated T cells in TCR junctional sequences. Int. Immunol..

[230] Wucherpfennig K., Strominger J. (1995). Molecular Mimicry in T-Cell-Mediated Autoimmunity–Viral Peptides Activate Human T-Cell Clones Specific for Myelin Basic-Protein. Cell.

[231] Mao Y., Lu C., Wang X., Xiao B. (2007). Induction of experimental autoimmune encephalomyelitis in Lewis rats by a viral peptide with limited homology to myelin basic protein. Exp. Neurol..

[232] Wucherpfennig K.W., Sette A., Southwood S., Oseroff C., Matsui M., Strominger J.L., Hafler D.A. (1994). Structural Requirements for Binding of an Immunodominant Myelin Basic-Protein Peptide to Dr2 Isotypes and for its Recognition by Human T-Cell Clones. J. Exp. Med..

[233] Warren K.G., Catz I., Steinman L. (1995). Fine Specificity of the Antibody-Response to Myelin Basic-Protein in the Central-Nervous-System in Multiple-Sclerosis–the Minimal B-Cell Epitope and a Model of its Features. Proc. Natl. Acad. Sci. USA.

[234] Smith K., Pyrdol J., Gauthier L., Wiley D., Wucherpfennig K. (1998). Crystal structure of HLA-DR2 (DRA*0101, DRB1*1501) complexed with a peptide from human myelin basic protein. J. Exp. Med..

[235] Li Y.L., Huang Y.P., Lue J., Quandt J.A., Martin R., Mariuzza R.A. (2005). Structure of a human autoimmune TCR bound to a myelin basic protein self-peptide and a multiple sclerosis-associated MHC class II molecule. EMBO J..

[236] Pfister H.W., Einhaupl K.M., Wick M., Fatehmoghadam A., Huber M., Schielke E., Goebel F.D., Matuschke A., Heinrich B., Bogner J.R. (1989). Myelin Basic-Protein in the Cerebrospinal-Fluid of Patients Infected with Hiv. J. Neurol..

[237] Albert L., Inman R. (1999). Mechanisms of disease: Molecular mimicry and autoimmunity. N. Engl. J. Med..

[238] D’Souza C., Wood D., She Y., Moscarello M. (2005). Autocatalytic cleavage of myelin basic protein: An alternative to molecular mimicry. Biochemistry.

[239] Pritzker L., Joshi S., Gowan J., Harauz G., Moscarello M. (2000). Deimination of myelin basic protein. 1. Effect of deimination of arginyl residues of myelin basic protein on its structure and susceptibility to digestion by cathepsin D. Biochemistry.

[240] Medveczky P., Antal J., Patthy A., Kekesi K., Juhasz G., Szilagyi L., Graf L. (2006). Myelin basic protein, an autoantigen in multiple sclerosis, is selectively processed by human trypsin 4. FEBS Lett..

[241] D’Souza C.A., Moscarello M.A. (2006). Differences in susceptibility of MBP charge isomers to digestion by stromelysin-1 (MMP-3) and release of an immunodominant epitope. Neurochem. Res..

[242] Williams K.R., Williams N.D., Konigsberg W., Yu R.K. (1986). Acidic Lipids Enhance Cathepsin-D Cleavage of the Myelin Basic-Protein. J. Neurosci. Res..

[243] Shaharabani R., Ram-On M., Avinery R., Aharoni R., Arnon R., Talmon Y., Beck R. (2016). Structural transition in myelin membrane as initiator of multiple sclerosis. J. Am. Chem. Soc..

[244] Shaharabani R., Ram-On M., Talmon Y., Beck R. (2018). Pathological transitions in myelin membranes driven by environmental and multiple sclerosis conditions. Proc. Natl. Acad. Sci. USA.

[245] Frankenhaeuser B. (1957). The effect of calcium on the myelinated nerve fibre. J. Physiol..

[246] Haak L.L., Grimaldi M., Russell J.T. (2000). Mitochondria in myelinating cells: Calcium signaling in oligodendrocyte precursor cells. Cell Calcium.

[247] Cheli V.T., Santiago González D.A., Namgyal Lama T., Spreuer V., Handley V., Murphy G.G., Paez P.M. (2016). Conditional Deletion of the L-Type Calcium Channel Cav1.2 in Oligodendrocyte Progenitor Cells Affects Postnatal Myelination in Mice. J. Neurosci..

[248] Krasnow A.M., Ford M.C., Valdivia L.E., Wilson S.W., Attwell D. (2018). Regulation of developing myelin sheath elongation by oligodendrocyte calcium transients in vivo. Nat. Neurosci..

[249] Miller R.H. (2018). Calcium control of myelin sheath growth. Nat. Neurosci..

[250] Friess M., Hammann J., Unichenko P., Luhmann H.J., White R., Kirischuk S. (2016). Intracellular ion signaling influences myelin basic protein synthesis in oligodendrocyte precursor cells. Cell Calcium.

[251] Paez P.M., Spreuer V., Handley V., Feng J.M., Campagnoni C., Campagnoni A.T. (2007). Increased expression of golli myelin basic proteins enhances calcium influx into oligodendroglial cells. J. Neurosci..

[252] Tsang D., Tsang Y.S., Ho W.K., Wong R.N. (1997). Myelin Basic Protein Is a Zinc-Binding Protein in Brain: Possible Role in Myelin Compaction. Neurochem. Res..

[253] Riccio P., Giovannelli S., Bobba A., Romito E., Fasano A., Bleve-Zacheo T., Favilla R., Quagliariello E., Cavatorta P. (1995). Specificity of zinc binding to myelin basic protein. Neurochem. Res..

[254] Weil M., Möbius W., Winkler A., Ruhwedel T., Wrzos C., Romanelli E., Bennett J.L., Enz L., Goebels N., Nave K. (2016). Loss of Myelin Basic Protein Function Triggers Myelin Breakdown in Models of Demyelinating Diseases. Cell Rep..

[255] Ramchandren S. (2017). Charcot-Marie-Tooth Disease and Other Genetic Polyneuropathies. Contin. (Minneap. Minn.).

[256] Roda R.H., McCray B.A., Klein C.J., Hoke A. (2018). Novel hemizygous nonsense mutation in DRP2 is associated with inherited neuropathy. Neurol. Genet..

[257] Datta S., Kataria S., Govindarajan R. (2019). A Case Report on Charcot-Marie-Tooth Disease with a Novel Periaxin Gene Mutation. Cureus.

[258] Takashima H., Boerkoel C., De Jonghe P., Ceuterick C., Martin J., Voit T., Schroder J., Williams A., Brophy P., Timmerman V. (2002). Periaxin mutations cause a broad spectrum of demyelinating neuropathies. Ann. Neurol..

[259] Otagiri T., Sugai K., Kijima K., Arai H., Sawaishi Y., Shimohata M., Hayasaka K. (2006). Periaxin mutation in Japanese patients with Charcot-Marie-Tooth disease. J. Hum. Genet..

[260] Guilbot A., Williams A., Ravise N., Verny C., Brice A., Sherman D., Brophy P., LeGuern E., Delague V., Bareil C. (2001). A mutation in periaxin is responsible for CMT4F, an autosomal recessive form of Charcot-Marie-Tooth disease. Hum. Mol. Genet..

[261] Marchesi C., Milani M., Morbin M., Cesani M., Lauria G., Scaioli V., Piccolo G., Fabrizi G.M., Cavallaro T., Taroni F. (2010). Four novel cases of periaxin-related neuropathy and review of the literature. Neurology.

[262] Nouioua S., Hamadouche T., Funalot B., Bernard R., Bellatache N., Bouderba R., Grid D., Assami S., Benhassine T., Levy N. (2011). Novel mutations in the PRX and the MTMR2 genes are responsible for unusual Charcot-Marie-Tooth disease phenotypes. Neuromusc. Disord..

[263] Boerkoel C., Takashima H., Stankiewicz P., Garcia C., Leber S., Rhee-Morris L., Lupski J. (2001). Periaxin mutations cause recessive Dejerine-Sottas neuropathy. Am. J. Hum. Genet..

[264] Choi Y.J., Hyun Y.S., Nam S.H., Koo H., Bin Hong Y., Chung K.W., Choi B. (2015). Novel Compound Heterozygous Nonsense PRX Mutations in a Korean Dejerine-Sottas Neuropathy Family. J. Clin. Neurol..

[265] Kabzinska D., Drac H., Sherman D., Kostera-Pruszczyk A., Brophy P., Kochanski A., Hausmanowa-Petrusewicz I. (2006). Charcot-Marie-Tooth type 4F disease caused by S399fsx410 mutation in the PRX gene. Neurology.

[266] Schabhüttl M., Wieland T., Senderek J., Baets J., Timmerman V., De Jonghe P., Reilly M.M., Stieglbauer K., Laich E., Windhager R. (2014). Whole-exome sequencing in patients with inherited neuropathies: Outcome and challenges. J. Neurol..

[267] Tokunaga S., Hashiguchi A., Yoshimura A., Maeda K., Suzuki T., Haruki H., Nakamura T., Okamoto Y., Takashima H. (2012). Late-onset Charcot-Marie-Tooth disease 4F caused by periaxin gene mutation. Neurogenetics.

[268] Auer-Grumbach M., Fischer C., Papic L., John E., Plecko B., Bittner R.E., Bernert G., Pieber T.R., Miltenberger G., Schwarz R. (2008). Two novel mutations in the GDAP and PRX genes in early onset Charcot-Marie-Tooth syndrome. Neuropediatrics.

[269] Nagase T., Kikuno R., Nakayama M., Hirosawa M., Ohara O. (2000). Prediction of the coding sequences of unidentified human genes. XVIII. The complete sequences of 100 new cDNA clones from brain which code for large proteins in vitro. DNA Res..

[270] Kijima K., Numakura C., Shirahata E., Sawaishi Y., Shimohata M., Igarashi S., Tanaka T., Hayasaka K. (2004). Periaxin mutation causes early-onset but slow-progressive Charcot-Marie-Tooth disease. J. Hum. Genet..

[271] Parman Y., Battaloglu E., Baris I., Bilir B., Poyraz M., Bissar-Tadmouri N., Williams A., Ammar N., Nelis E., Timmerman V. (2004). Clinicopathological and genetic study of early-onset demyelinating neuropathy. Brain.

[272] Sherman D.L., Brophy P.J. (2018). A murine model of Charcot-Marie-Tooth disease 4F reveals a role for the C-terminus of periaxin in the formation and stabilization of Cajal bands. Wellcome Open Res..

[273] Renouil M., Stojkovic T., Jacquemont M.L., Lauret K., Boue P., Fourmaintraux A., Randrianaivo H., Tallot M., Mignard D., Roelens P. (2013). Charcot-Marie-Tooth disease associated with periaxin mutations (CMT4F): Clinical, electrophysiological and genetic analysis of 24 patients. Rev. Neurol..

[274] Barankova L., Siskova D., Hühne K., Vyhnalkova E., Sakmaryova I., Bojar M., Rautenstrauss B., Seeman P. (2008). A 71-nucleotide deletion in the periaxin gene in a Romani patient with early-onset slowly progressive demyelinating CMT. Eur. J. Neurol..

[275] Konrat R. (2014). NMR contributions to structural dynamics studies of intrinsically disordered proteins. J. Magn. Reson..

[276] Wallace B.A. (2019). The role of circular dichroism spectroscopy in the era of integrative structural biology. Curr. Opin. Struct. Biol..

[277] Kikhney A.G., Svergun D.I. (2015). A practical guide to small angle X-ray scattering (SAXS) of flexible and intrinsically disordered proteins. FEBS Lett..

[278] Schuler B., Muller-Spath S., Soranno A., Nettels D. (2012). Application of confocal single-molecule FRET to intrinsically disordered proteins. Methods Mol. Biol..

[279] Baul U., Chakraborty D., Mugnai M.L., Straub J.E., Thirumalai D. (2019). Sequence Effects on Size, Shape, and Structural Heterogeneity in Intrinsically Disordered Proteins. J. Phys. Chem. B.

[280] Ghosh I., Considine N., Maunus E., Sun L., Zhang A., Buswell J., Evans T.C., Xu M. (2011). Site-Specific Protein Labeling by Intein-Mediated Protein Ligation. Methods Mol. Biol..

[281] Stevens A.J., Sekar G., Shah N.H., Mostafavi A.Z., Cowburn D., Muir T.W. (2017). A promiscuous split intein with expanded protein engineering applications. Proc. Natl. Acad. Sci. USA.

[282] Binder H., Arnold K., Ulrich A.S., Zschornig O. (2001). Interaction of Zn2+ with phospholipid membranes. Biophys. Chem..

[283] Gillooly D., Simonsen A., Stenmark H. (2001). Cellular functions of phosphatidylinositol 3-phosphate and FYVE domain proteins. Biochem. J..

