# Correction: Raasakka, A.; Kursula, P. Flexible Players within the Sheaths: The Intrinsically Disordered Proteins of Myelin in Health and Disease. *Cells* 2020, *9*, 470

**DOI:** 10.3390/cells11040662

**Published:** 2022-02-14

**Authors:** Arne Raasakka, Petri Kursula

**Affiliations:** 1 The Department of Biomedicine, University of Bergen, Jonas Lies vei 91, NO-5009 Bergen, Norway; arne.raasakka@uib.no; 2 Faculty of Biochemistry and Molecular Medicine & Biocenter Oulu, University of Oulu, Aapistie 7A, FI-90220 Oulu, Finland

The authors would like to make corrections to their review [1] in the light of two recently retracted papers [2,3]. These papers (citations 202 and 203 in the original publication) were cited and the information within was discussed in our review, as well as used in the preparation of Figure 8. We have now removed these citations and all information from the two retracted papers by modifying our review text, as described below.

The following text from Section 2.7 has been updated by replacing citation 202 with citation 200: “The interaction is thought to be mainly mediated by the DRP2 spectrin repeat domain, with possible involvement of the adjacent WW domain and NLS2/NLS3 in L-PRX (Figure 8b) [13,202]”.

The following text from Section 2.7 has been deleted: “The NLS2/NLS3 region mediates a self-interaction within L-PRX by binding to the L-PRX acidic C terminus, although it is unclear whether the interaction is intramolecular or if two L-PRX molecules may interact [203]”.

The following text from Section 2.7 has been deleted: “The C-terminal acidic region is rich in Glu, which is believed to be the basis of the interaction with NLS2 and NLS3 of the basic region (Figure 8b). The collective negative charge is likely to play a profound role in the association, although one specific point mutation, E1259K, had a significant inhibitory effect [203]”.

The following text from the end of paragraph eight in Section 2.7 has been deleted: “(*see below*)”.

The following text from Section 2.7 has been deleted: “Finally, the self-association of the acidic domain with the tripartite NLS might harbor similar roles in either nuclear export or inhibition of DRP2 and/or integrin β4 binding [203]. It remains to be determined, whether the mechanism involves two interacting L-PRX molecules or an intramolecular switch within a single L-PRX molecule that concurrently forms hetero- and homodimers via the PDZ domain”.

The following paragraph in Section 2.7 has been deleted: “Bringing several L-PRX molecules together through the aforementioned interactions could result in large-scale assemblies that undergo liquid–liquid phase separation. This could harbor a regulatory role in the formation and stability of the periaxinosome, for instance through the controlled release of free L-PRX to the cytosol or to the membrane surface [216]. The binding of ezrin to L-PRX, or S-PRX heterodimerization with L-PRX, might function as a control mechanism for the formation of phase-separated compartments in the periaxinosome”.

The following text from Section 3.2 has been deleted: “The only missense mutation that has a known effect is E1259K, which abolishes the internal L-PRX interaction [203]”.

The following text from Section 4 “The intramolecular interaction within L-PRX and the potential regulatory role of S- and L-PRX heterodimerization need to be characterized at the molecular level, in order to shed light on PRX nuclear trafficking and the formation of the periaxinosome.” has been modified to: “The potential regulatory role of S- and L-PRX heterodimerization needs to be characterized at the molecular level, in order to shed light on PRX nuclear trafficking and the formation of the periaxinosome”.

Figure 8 has been modified. The updated figure and figure legend are shown below.

**Figure 8:**
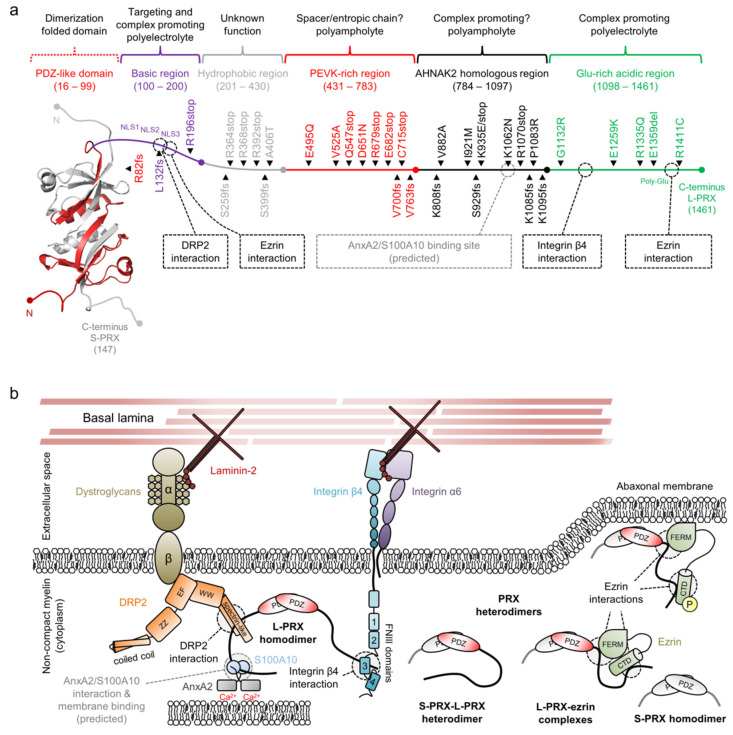
The structure and interactions of periaxin. (**a**) A schematic of a PRX heterodimer, with S-PRX in gray and L-PRX colored based on region, with the PDZ domain in red. L-PRX, apart from the PDZ-like domain, is predicted to be disordered [45], and can be divided into separate regions based on sequence composition. Peripheral neuropathy mutations are indicated alongside L-PRX. Dashed boxes and lines denote protein–protein interactions. L-PRX contains a predicted AnxA2 and S100A10 binding region, as reported earlier for AHNAK [195,196]. See Table 1 for mutation details. (**b**) L-PRX is an assembler within abaxonal non-compact myelin, linking dystroglycans and integrins together in membrane appositions, forming the periaxinosome. These interaction partners connect the Schwann cell basal lamina to the Schwann cell cytoplasm. S-PRX forms heterodimers with L-PRX, which might allow regulation of the cytoplasmic assembly as well as the nuclear export of L-PRX. Ezrin in complex with hetero- or homodimeric L-PRX might have relevance in such regulations, especially considering its phosphoregulated membrane-binding activity [197]. The function of the S-PRX homodimer is unknown. The significance of the putative L-PRX/AnxA2/Sl00A10 ternary complex could involve linking the entire assembly via AnxA2 and Ca^2+^ to the underlying membrane, possibly forming a structural basis for membrane appositions that line Cajal bands in myelinating Schwann cells.

Table 1 has been modified. The updated table is shown below.

**Table 1:** PRX mutations, related neuropathies and potential molecular mechanisms.

Mutation ^1^	Neuropathy	(Potential) Molecular Impact	Reference(s)
R82fs	DSS	Tail loss; loss of interactions	[258]
L132fs	CMT4F	Tail loss; loss of interactions	[259]
R196stop	CMT4F		[260]
S259fs	CMT4F	Loss of hydrophobic, PEVK-rich, AHNAK2 homology and acidic regions; loss of interactions	[261]
R364stop	CMT4F	Loss of PEVK-rich, AHNAK2 homology and acidic regions; loss of interactions	[262]
R368stop	DSS	Loss of PEVK-rich, AHNAK2 homology and acidic regions; loss of interactions	[263]
R392stop	DSS	Loss of PEVK-rich, AHNAK2 homology and acidic regions; loss of interactions	[264]
S399fs	CMT4F	Loss of PEVK-rich, AHNAK2 homology and acidic regions; loss of interactions	[265]
A406T	DSS		[263]
E495Q	DSS		[263]
V525A	CMT4F		[260,266]
Q547stop	CMT4F	Loss of PEVK-rich (partial), AHNAK2 homology and acidic regions; loss of interactions	[261]
D651N	CMT4F		[267]
R679stop	DSS	Loss of PEVK-rich (partial), AHNAK2 homology and acidic regions; loss of interactions	[264]
E682stop	CMT4F	Loss of PEVK-rich (partial), AHNAK2 homology and acidic regions; loss of interactions	[261]
A700fs	CMT4F		[268]
C715stop	DSS	Loss of PEVK-rich (partial), AHNAK2 homology and acidic regions; loss of interactions	[258]
V763fs	DSS	Loss of PEVK-rich (partial), AHNAK2 homology and acidic regions; loss of interactions	[263]
K808fs	CMT4F	Loss of AHNAK2 homology and acidic regions; loss of interactions	[261]
V882A	DSS		[263,269]
I921M	DSS		[263]
S929fs	DSS	Loss of AHNAK2 homology and acidic regions; loss of interactions	[263]
K935E	DSS		[263]
K935stop	DSS	Loss of acidic domain; loss of integrin interaction	[263]
K1062N	CMT4F	(Loss of predicted AnxA2/S100A10 interaction?)	[257]
R1070stop	CMT4F	Loss of acidic domain; loss of integrin interaction	[208,259,267,270–272]
P1083R	DSS		[265]
E1085fs	CMT4F	Loss of acidic domain; loss of integrin interaction	[273]
K1095fs	CMT4F	Loss of acidic domain; loss of integrin interaction	[274]
G1132R	DSS		[263]
E1259K	DSS		[263]
R1335Q ^2^	CMT		[266]
E1359del	DSS		[263]
R1411C	DSS		[263]

^1^ fs denotes frame shift mutation, stop denotes nonsense mutation. ^2^ Found together with V525A in a complex neuropathy associated with dysarthria, hypermobile joints, and cerebellar symptoms.

The authors apologize for any inconvenience caused. The original article has been updated.
